# Tough Cellulose Hydrogel Electrolyte with Low Solvation for Highly Reversible and Flexible Aqueous Zinc‐Ion Battery

**DOI:** 10.1002/advs.202511759

**Published:** 2025-07-29

**Authors:** Fan Chen, Xuan Li, Shi‐Peng Chen, Yilin Zhang, Hua‐Dong Huang, Hongli Yang, Shengyang Zhou, Zhong‐Ming Li

**Affiliations:** ^1^ College of Materials Science and Engineering Sichuan University Chengdu 610065 China; ^2^ College of Polymer Science and Engineering Sichuan University Chengdu 610065 China; ^3^ West China Hospital/West China School of Medicine Sichuan University Chengdu 610041 China; ^4^ State Key Laboratory of Advanced Polymer Materials Sichuan University Chengdu 610065 China

**Keywords:** aqueous battery, cellulose hydrogel, hydrogel electrolyte, ion coordination structure regulation

## Abstract

Recent advancements in hydrogel electrolytes for aqueous zinc‐ion batteries (AZIBs) have drawn considerable interest due to their soft nature, offering potential to overcome challenges including reversibility and flexibility. As the most abundant natural polymer, cellulose is ideal for AZIB hydrogel electrolytes due to rich hydroxyls with stable hydrogen‐bonded networks for water retention. However, conventional cellulose hydrogels suffer from low Zn^2+^ conductivity and insufficient mechanical robustness, usually requiring additional polymers to meet practical demands. This work reports a chemically neutral dissolution system combined with Keggin‐type polyoxometalate as a bifunctional crosslinker and electrolyte modulator. This approach results in ultra‐low solvation of Zn^2+^ in cellulose hydrogel, contributing to a wide 2.48 V electrochemical stability window. The high‐desolvation hydrogel exhibits balanced Zn^2+^ reaction stability and transport kinetics, effectively suppressing dendrite growth and parasitic reactions. The Zn electrode can be stably strapped/plated with this hydrogel for thousands of cycles with minimal Coulomb efficiency change. The hydrogel also shows excellent flexibility, with toughness of 1.5 MJ m^−3^ and elongation at break of 80%. Pouch cells assembled with this hydrogel demonstrate high mechanical flexibility and stability under deformations. This pioneering cellulose dissolution and crosslinking chemistry paves the way for practical application of flexible, durable AZIBs.

## Graphical abstract

Polyoxometalate‐crosslinked cellulose hydrogel enables efficient Zn^2+^ desolvation, dendrite suppression, and mechanical flexibility, offering a robust electrolyte for aqueous zinc‐ion batteries.

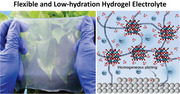

## 1 Introduction

Aqueous zinc‐ion batteries (AZIBs) have emerged as promising candidates for next‐generation wearable power sources due to their inherent safety, environmental friendliness, and low manufacturing cost.^[^ ^1^, ^2^, ^3^ ^]^ The use of aqueous electrolytes avoids the toxicity, flammability, and volatility associated with organic systems, while offering a safer alternative for skin‐mounted or implantable devices operating within the human physiological environment.^[^ ^4^, ^5^, ^6^ ^]^ Additionally, AZIBs provide moderate to high energy density, meeting the increasing demand for miniaturized and durable power systems in wearable applications. Their compatibility with abundant and non‐toxic materials further enhances sustainability and scalability.^[^ ^7^, ^8^ ^]^ Despite these advantages, the practical deployment of AZIBs in wearable electronics remains limited by key challenges, including poor cycling reversibility, uncontrolled zinc dendrite formation, and limited mechanical integrity of battery components under deformation.^[^ ^9^, ^10^ ^]^ These issues compromise device reliability, operational lifespan, and user safety during repeated mechanical stress. Moreover, parasitic reactions at the zinc‐electrolyte interface and narrow electrochemical stability windows hinder the development of long‐lasting systems.^[^ ^11^, ^12^, ^13^, ^14^ ^]^ Addressing these constraints requires a deeper understanding of electrolyte‐electrode interactions, improved control over zinc‐ion transport and deposition behavior, and the development of stable, mechanically resilient architectures. Overcoming these technical barriers is essential for advancing AZIBs toward flexible, high‐performance energy storage solutions suited for next‐generation wearable technologies.

Hydrogels are entangled hydrophilic polymer networks that retain large amounts of water while maintaining structural integrity.^[^ ^15^, ^16^ ^]^ They offer tissue‐like softness, high biocompatibility, tunable mechanics, and ion conduction. In addition, the soft and deformable structure of hydrogels enables mechanical adaptability under complex wearable conditions. Moreover, the tunable architecture of hydrogels allows for targeted regulation of Zn^2^⁺ transport, which benefits the suppression of Zn dendrite formation while improving cycling stability.^[^ ^17^, ^18^ ^]^ These inherent advantages collectively position hydrogel electrolytes as promising components for the development of flexible and stable AZIBs, especially for wearable electronics. As the most abundant renewable biopolymer on Earth, cellulose possesses intrinsic biocompatibility and dense hydroxyl functionality that enables strong hydrogen‐bonded networks for water retention, making it a promising matrix for hydrogel electrolytes for AZIBs.^[^ ^19^, ^20^ ^]^ However, conventional cellulose‐based hydrogels suffer from inadequate mechanical resilience for wearable applications and exhibit unsatisfactory electrochemical performance due to limited Zn^2^⁺ conductivity and uncontrolled parasitic reactions. These limitations stem from the low solubility and harsh alkaline conditions required in traditional dissolution systems, which result in relatively low cellulose solid content and degraded molecular weight, compromising the toughness and strength of the obtained hydrogels.^[^ ^21^, ^22^ ^]^ The current reported cellulose hydrogels usually rely on high‐molecular‐weight polymer additives to meet mechanical requirements for battery electrolyte use.^[^ ^23^, ^24^ ^]^ Moreover, the lack of control over the coordination environment within the cellulose hydrogel electrolyte restricts the modulation of Zn^2^⁺ solvation structures, which leads to the difficulty of suppressing parasitic reactions such as hydrogen evolution and zinc hydroxide formation, ultimately hindering the reversibility and stability of AZIBs. Improving the solid content of cellulose without compromising its molecular integrity is essential to enhancing the mechanical strength and flexibility of cellulose‐based hydrogel electrolytes. In parallel, implementing coordination environment regulation within the electrolyte is likewise vital for suppressing parasitic reactions and directing uniform zinc deposition, which is also key to achieving long‐term electrochemical cycling stability.

**Scheme 1:**
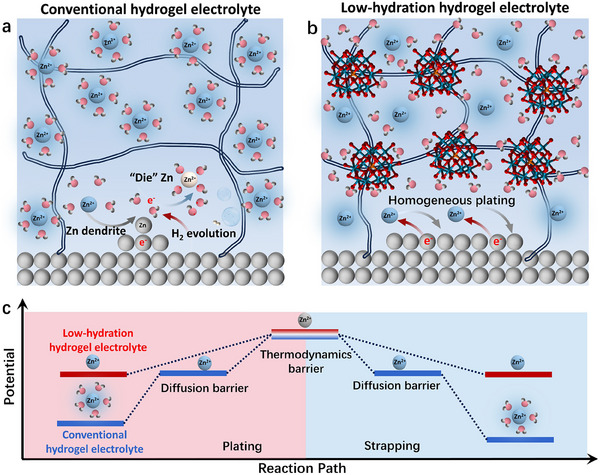
Mechanistic illustration of Zn deposition behavior at the interface of anode with a) conventional hydrogel electrolyte and b) low‐hydration hydrogel electrolyte. c) Potential fluctuation of zinc ions in low‐hydration and conventional hydrogels during electrochemical plating and strapping process.

In this study, a chemically neutral dissolution system has been developed to efficiently dissolve natural cellulose without degrading its molecular structure. A polyoxometalate crosslinker with a Keggin‐type configuration (silicotungstic zinc) was employed to construct a physically crosslinked network. After incorporating 2 M ZnSO_4_, the resulting hydrogel electrolyte (STA‐hydrogel) exhibited outstanding mechanical performance, including a tensile strength exceeding 3 MPa, toughness of 1.5 MJ m^−^^3^, and elongation at break over 80%, surpassing most reported pure regenerated cellulose hydrogels. More importantly, the polyoxometalate can effectively reduce the solvation of Zn^2^⁺, while maintaining the rapid Zn^2^⁺ transport kinetics (**Scheme** **1**a,b), which enabled a wide electrochemical stability window up to 2.48 V and supported a high Zn^2^⁺ conductivity of 22 mS m^−^^1^. Electrochemical measurements revealed an optimized electrochemical thermodynamic path and stability of STA‐hydrogel (Scheme 1c), which enables the effective suppression of parasitic reactions and promotion of uniform planar zinc deposition. The Zn electrode delivered stable plating and stripping reversibility over 3000 h when paired with STA‐hydrogel. By using V_2_O_5_ as a cathode, the full cell maintained a capacity of more than 200 mAh g^−^^1^ over 1000 cycles at a current density of 0.5 A g^−^^1^. Additionally, soft‐packaged cells based on STA‐hydrogel retained stable performance under severe mechanical bending and deformation.

## 2 Results and Discussion

**Figure 1:**
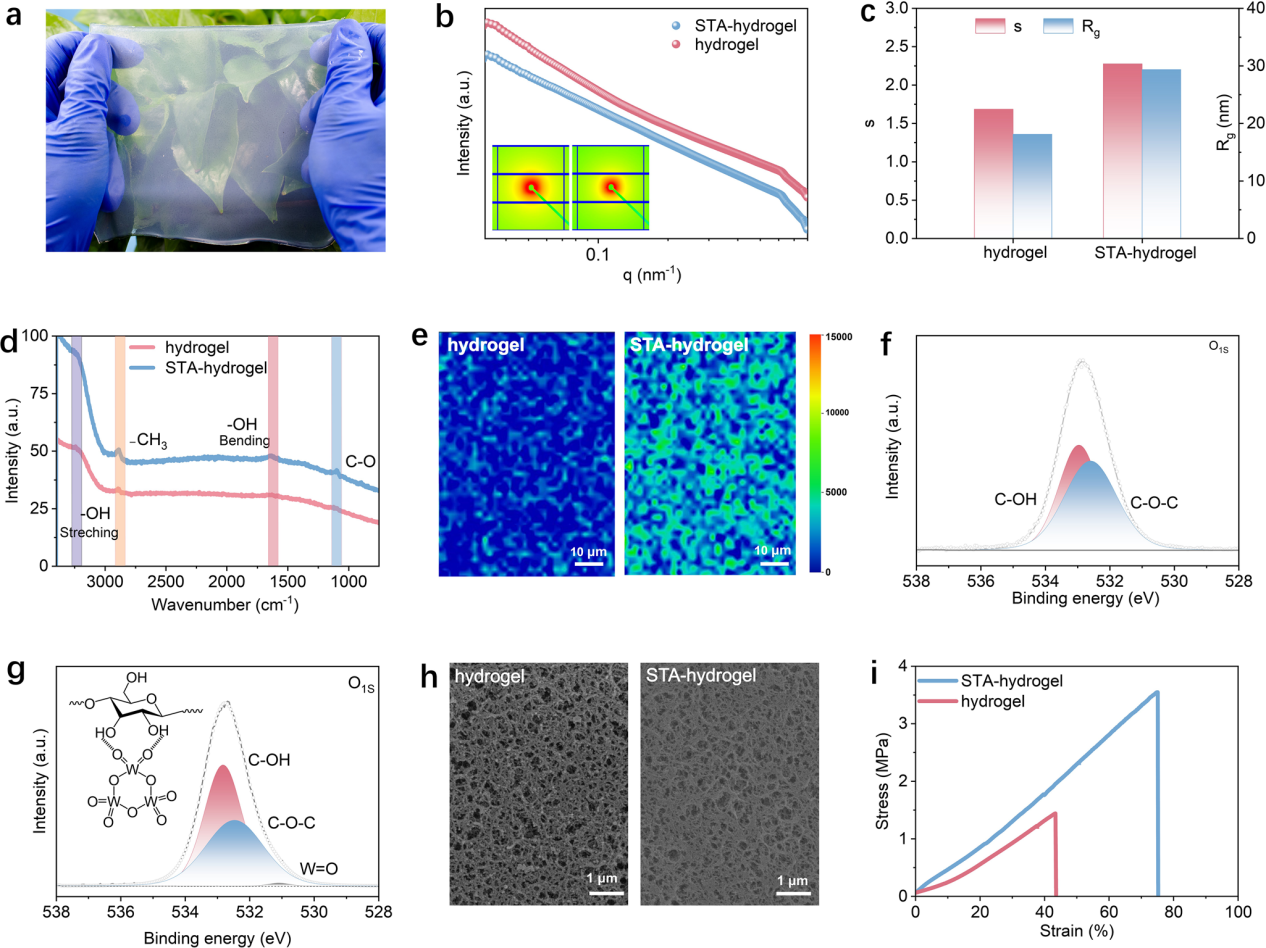
Structural characterization of pristine cellulose and STA hydrogel electrolytes. a) Optical photo of STA hydrogel with a size of 20 cm × 20 cm. b) 2D SAXS patterns (inset) and corresponding 1D SAXS intensity curves of two hydrogels. c) The corresponding size parameters obtained by SAXS fitting. d) Raman spectra of pristine hydrogel and STA hydrogel. e) Raman mapping images of two hydrogels. f) XPS spectra of O_1s_ for pristine hydrogel. g) XPS spectra of O_1s_ for STA hydrogel and XPS spectra of O_1s_ for pristine hydrogel. h) Surface FE‐SEM images of two hydrogels pre‐dried by CPD and APD in advance. i) Stress–strain curves of pristine hydrogel and SAT hydrogel.

The STA hydrogel electrolyte was facially prepared via a straightforward and scalable method. Commonly used cotton fiber or lab filter paper can be used as the cellulose source and dissolved in a pH‐neutral binary solvent system (TMG/ MAA, mass ratio 1:1). A small amount of tungstosilicic acid hydrate (≈0.1 wt%) was then introduced to cross‐link the cellulose molecular chains. The resulting cellulose solution remained clear and homogeneous, with significantly increased viscosity, indicating effective crosslinking (Figure S1, Supporting Information). The cellulose was regenerated using water as the coagulation bath to obtain hydrogel, followed by ion exchange to achieve an internal ZnSO_4_ concentration to 2 mol L^−1^. This method enables the facile fabrication of large‐sized hydrogel electrolytes (**Figure** **1**a) with uniform distribution of STA (Figure S2, Supporting Information) and tailored thickness. In this study, a 200 µm‐thick STA hydrogel electrolyte was prepared to meet the demands of high‐energy‐density battery devices (Figure S2, Supporting Information). X‐ray small‐angle scattering (SAXS) reveals that the STA‐hydrogel exhibits reduced intensity and sharper Kratky features, indicating a more compact and ordered network compared to that of pristine cellulose hydrogel (Figure 1b). Higher values of calculated scattering exponent *s* and radius of gyration *R_g_* for STA‐hydrogel suggest enhanced segmental correlation and larger structural domains (Figure 1c; Figure S3, Supporting Information), confirming that STA crosslinking reinforces network organization. Furthermore, we investigated the molecular‐level structural changes of cellulose hydrogels upon STA incorporation by Raman spectroscopy (Figure 1d). After crosslinking, the Raman bands of both the stretching and bending vibrations of hydroxyl groups increase compared to that of pristine cellulose. This enhancement arises from the highly polarized metal‐oxygen bonds of the polyoxometalate, which interacts with hydroxyls through a combination of hydrogen bonding and coordination. These interactions intensify the local polarizability changes around hydroxyl groups, thereby amplifying the Raman signal. This result supports a crosslinking mechanism driven by strong hydrogen bonding and coordination effects of STA. Spatial Raman mapping of the hydroxyl bending vibrations further confirmed that this interaction is uniformly distributed throughout the hydrogel matrix rather than localized interaction (Figure 1e).

In addition, the results of X‐ray photoelectron spectroscopy (XPS) provide additional evidence of this crosslinking mechanism (Figures S4 and S5, Supporting Information). Upon STA crosslinking, the C─O─H band shifts to lower binding energy with increased intensity, suggesting a strong electronic interaction between cellulose hydroxyls and the metal‐oxygen bonds in STA (Figure 1f,g). This interaction likely involves partial electron delocalization from oxygen lone pairs, which reduces the electron‐withdrawing effect on adjacent carbon atoms and increases the local electron density, thereby stabilizing the hydroxyl's environment. As a result, this distinctive crosslinking effect promotes a more uniform and compact fibrillar structure within the cellulose hydrogel (Figure 1h), which can also be confirmed by the results of energy‐dispersive X‐ray spectroscopy (EDS) (Figures S6 and S7, Supporting Information). After crosslinking, the STA hydrogel shows improved water retention capacity compared to the pristine cellulose hydrogel (Figure 1c; Figure S8, Supporting Information). This enhanced ability to retain water helps preserve electrolyte continuity and ion mobility, thereby ensuring stable electrochemical performance over time. The mechanical performance is also significantly enhanced (Figure 1i), with a tensile strength up to 3.5 MPa, elongation at break approaching 80%, and toughness of 1.5 MJ m^−3^. These values markedly exceed those of the pristine cellulose hydrogel (Figure S9, Supporting Information) and previously reported cellulose‐based hydrogel electrolytes (Table S4, Supporting Information). This mechanical robustness not only ensures structural integrity under mechanical deformation but also supports stable ion transport under stress, which is essential for long‐term cycling stability and the practical realization of wearable or flexible devices.

Moreover, to clarify the role of crosslinking density, we systematically investigated its influence on both mechanical properties and Zn^2^⁺ conductivity (Figure S10, Supporting Information). Two key crosslinking mechanisms were considered: hydrogen bonding between cellulose chains, which is dependent on the solid content of cellulose, and supramolecular interactions between STA and cellulose, determined by STA concentration. Increasing the cellulose content leads to enhanced tensile strength and elongation due to denser hydrogen bonding networks. However, this also results in a reduced Zn^2^⁺ conductivity, likely caused by decreased free water and more restricted ion pathways within the compact hydrogel structure. Similarly, increasing STA concentration likewise improves mechanical strength through supramolecular reinforcement, but an excessive amount of STA decreases Zn^2^⁺ conductivity, which may also stem from reduced the ions activity caused by increased crosslinking. We further evaluated the swelling ratios of hydrogel electrolytes with different crosslinking densities. Here, the swelling ratio refers to the volumetric expansion from the dry state to the fully swollen hydrogel, as shown in Figure S11 (Supporting Information). The results reveal an overall negative correlation between swelling ratio and crosslinking degree. Specifically, increasing the cellulose solid content and STA concentration leads to a denser polymer network, resulting in lower swelling ratios. This trend confirms that both hydrogen bonding between cellulose chains and supramolecular interactions with STA contribute to higher crosslinking density. However, as the crosslinking density increases, the Zn^2^⁺ conductivity decreases significantly. This reduction is primarily attributed to restricted ion transport pathways and reduced water content, both of which limit ion mobility. Based on the combined analysis of mechanical and electrochemical performance, we selected a formulation of 3 wt% cellulose and 0.1 wt% STA for this work. This composition provides a balanced combination of mechanical strength, flexibility, and ionic conductivity. It ensures efficient Zn^2^⁺ transport while maintaining adequate mechanical integrity, which is critical for the reliable operation of flexible AZIBs.

**Figure 2:**
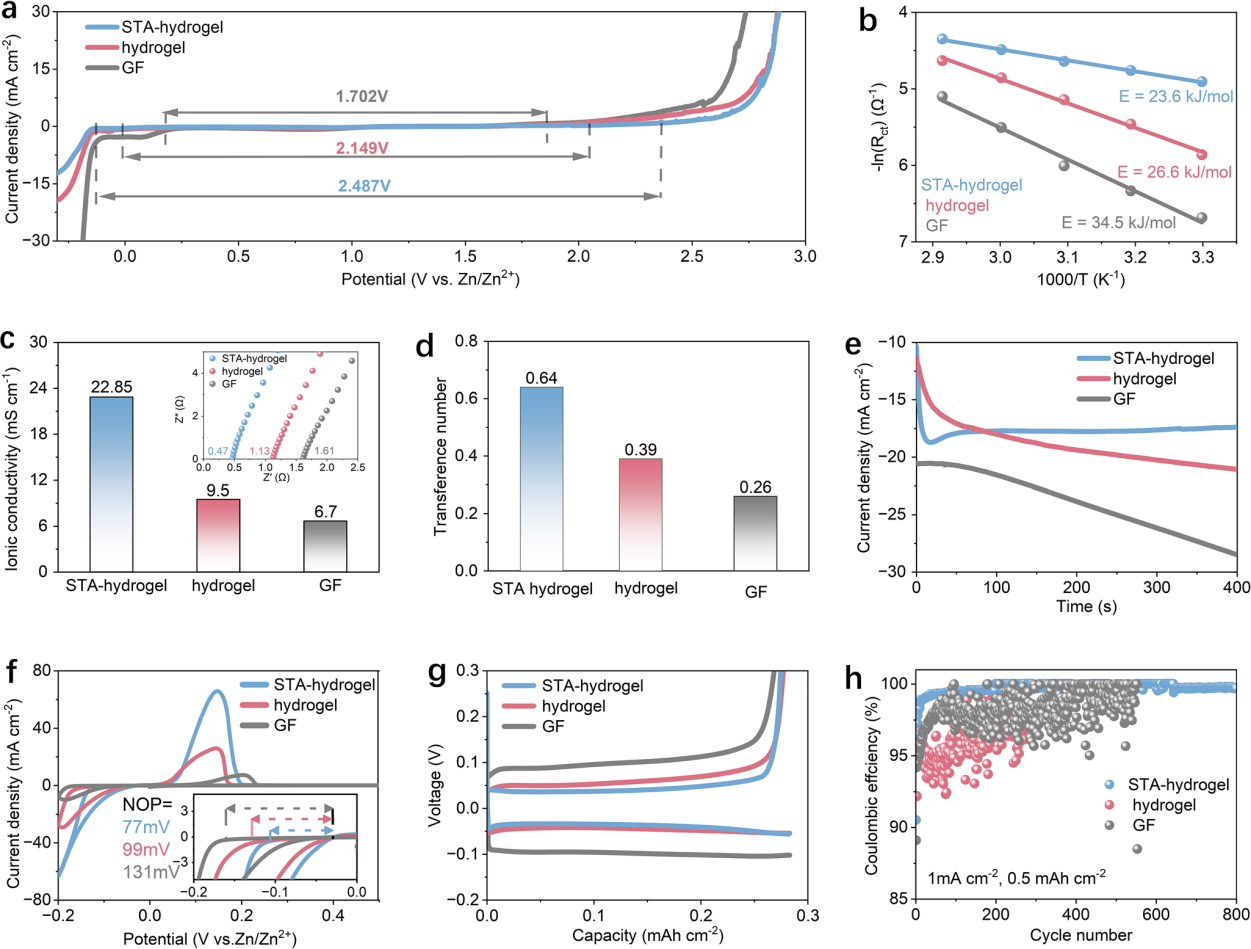
Electrochemical characterization of pristine cellulose and STA hydrogel electrolytes. a) The LSV curves of STA hydrogel electrolyte, pristine hydrogel electrolyte, and liquid electrolyte at 1 mV s^−1^ using stainless steel as electrodes. b) The activation energy fitting curves of three electrolytes. c) Ionic conductivity and EIS plots (inset) of three electrolytes, respectively. d) Zn^2+^ transference number of three electrolytes. e) The CA curves of Zn||Zn symmetric cells with three electrolytes under the bias voltage of −150 mV. f) CV curves of Zn||Cu asymmetric cells with three electrolytes at 1 mV s^−1^. g) Corresponding voltage‐capacity curves of Zn||Cu asymmetric cells with three electrolytes at 50 cycles. h) The coulombic efficiency of Zn||Cu asymmetric cells with three electrolytes, respectively.

The electrochemical properties of the cellulose hydrogel electrolytes before and after crosslinking, as well as the liquid electrolyte, were comparatively evaluated through a series of standardized measurements. Linear sweep voltammetry (LSV) measurements reveal that the electrochemical stability window of the STA hydrogel electrolyte reaches 2.487 V, significantly broader than that of the pristine cellulose hydrogel (2.149 V) and the liquid electrolyte (1.702 V) (**Figure** **2**a). This expansion suggests a disruption or weakening of the water hydrogen‐bonding network within the STA hydrogel, altering the local water structure and dielectric environment, which suppresses proton and electron transfer and thus elevates the water decomposition potential. Notably, despite this structural perturbation, the STA hydrogel exhibits a reduced Arrhenius activation energy compared to both the pristine cellulose hydrogel and the liquid electrolyte (Figure 2b; Figure S12, Supporting Information), along with an enhanced Zn^2+^ conductivity and improved transfer number (Figure 2c,d; Figure S13, Supporting Information). While conventional views suggest that weakening water structure or reducing free water typically impedes Zn^2^⁺ transport due to restricted solvation exchange,^[^ ^14^, ^25^ ^]^ the STA hydrogel presents yet a distinct behavior. The strong metal‐oxygen bonds at the STA surface induce Coulombic attraction with water protons, leading to a reorganization of water molecules. This restructures the hydration environment, weakens Zn^2+^ solvation, and enriches oxygen coordination sites, facilitating the formation of directional structural water channels that lower the diffusion barrier and enhance ionic mobility. This unique mechanism is closely related to the superchaotropic effect and is driven by the strong electrostatic attraction between polyoxometalates and water protons,^[^ ^26^, ^27^ ^]^ resulting in a combination of reduced Zn^2+^ solvation degree and improved transport kinetics. This phenomenon is actually not commonly observed in previously reported aqueous electrolytes or hydrogel electrolytes for AZIBs.

As a result, the STA hydrogel electrolyte with reduced Zn^2+^ solvation and enhanced ion transport properties enables stable and uniform zinc deposition. Chronoamperometry (CA) measurements show a consistent current response over a long period, indicating the effective suppression of unstable nucleation behavior and random dendritic growth (Figure 2e). Furthermore, the zinc electrode exhibits a higher deposition current and a more positive deposition potential when paired with STA hydrogel (Figure 2f), reflecting lower energy barriers for zinc nucleation and growth, thus improving the electrochemical reaction activity. In the tests of Zn─Cu asymmetric cell, the reduced overpotential observed during galvanostatic cycling confirms the enhanced kinetics and reversibility of zinc plating and stripping processes (Figure 2g; Figure S14, Supporting Information). To further elucidate the detailed mechanism of STA hydrogel‐mediated zinc deposition, we measured the interfacial Zn^2^⁺ diffusion coefficient as shown in Figure S15 (Supporting Information). The Zn^2^⁺ diffusion coefficient in the STA hydrogel electrolyte is nearly twice that of the conventional aqueous electrolyte, indicating significantly enhanced ion transport at the electrode interface. This improvement can likewise be attributed to the ability of STA to weaken the Zn^2+^ solvation structure and reorganize the hydrogen‐bonding network of water molecules, thereby reducing the energy barrier for interfacial migration. In addition, finite element analysis (FEA) was also performed to simulate the distribution of electric potential and current density near the zinc electrode in the presence of the STA hydrogel (Figure S16, Supporting Information). The simulation results reveal that the STA hydrogel not only promotes a more uniform interfacial electric field and current distributions, but also significantly reduces the potential difference across the electrode surface and minimizes the spatial variation in current density. These effects work in concert to lower the local driving force for uneven Zn nucleation, thereby reducing the interfacial nucleation barrier. This modulation of the electrochemical microenvironment provides an electrochemical basis for the enhanced uniformity and stability of zinc deposition in the STA system.

These favorable electrochemical characteristics are strongly linked to the altered solvation environment of Zn^2+^ within the STA hydrogel. A weakened solvation shell allows for faster desolvation at the electrode interface, facilitating more efficient charge transfer. Meanwhile, directional water networks within the hydrogel promote continuous ion transport with reduced diffusion resistance. Together, these effects lead to lower electrochemical polarization and more uniform Zn deposition. The modified solvation environment also limits free water and proton activity, which suppresses parasitic reactions such as hydrogen evolution and the formation of insulating zinc hydroxide species. Consequently, the STA hydrogel electrolyte enables Zn metal electrode with obviously improved Coulombic efficiency and long‐term cycling stability when compared to that of pristine cellulose hydrogel and conventional aqueous electrolytes (Figure 2h). Furthermore, STA‐based Zn─Cu cells can stably operate over 2000 cycles without short circuit (Figure S14, Supporting Information), demonstrating the robustness and reliability of the STA hydrogel electrolyte.

**Figure 3:**
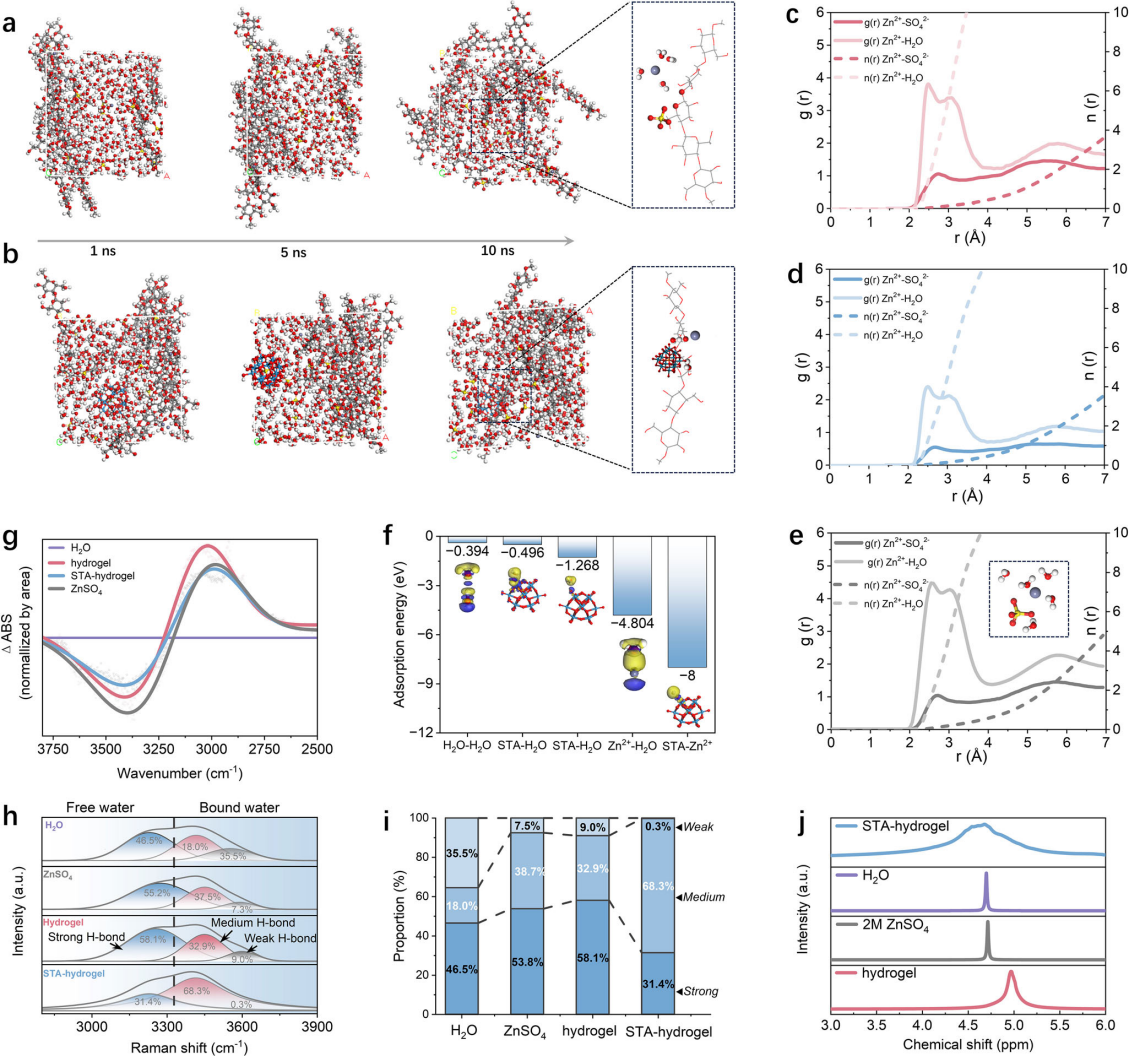
Comparative investigation of chemical coordination structures of STA hydrogel electrolytes. a) The MD simulation result of pristine hydrogel electrolyte. b) The MD simulation result of STA hydrogel electrolyte. Corresponding RDF plots of interaction between Zn^2+^ and other radicals in c) pristine hydrogel electrolyte d) STA hydrogel electrolyte, and e) liquid electrolyte. f) The adsorption energies between different components in the STA hydrogel, and corresponding charge density difference diagrams (inset). g) ATR‐FTIR spectra of different electrolytes. h) Raman spectra fitting of different electrolytes. i) the corresponding proportions of hydrogen bonds. j) ^1^H NMR spectra of different electrolytes.

To further elucidate the unique low‐solvation environment and rapid Zn^2+^ transport kinetics in the STA hydrogel, we performed a comparative investigation using molecular dynamics (MD) simulations including STA hydrogel, pristine cellulose hydrogel, and liquid electrolyte. In MD simulations, we found that the pristine cellulose hydrogel exhibits time‐dependent swelling and chain extension driven by the progressive infiltration of water. This process is governed by the disruption of intramolecular hydrogen bonds and the formation of new hydrogen bonds with surrounding water molecules, reflecting a pronounced structural relaxation and water uptake (**Figure** **3**a). In contrast, the STA hydrogel shows constrained chain dynamics due to electrostatic and coordination interactions between STA and hydroxyl groups on cellulose molecular chains, resulting in less localized and directional swelling behavior (Figure 3b). Water can still permeate the network, but the overall structural framework remains more stable, giving rise to stable hydration pathways and enhanced mechanical integrity and ionic conductivity. Coordination statistics from the MD trajectories reveal that the average Zn^2+^ hydration number decreases from g(r) ≈ 5 in 2 M ZnSO_4_ solution to g(r) ≈ 4 in pristine cellulose hydrogel and further to g(r) ≈ 2 in the STA hydrogel (Figure 3c–e). The underlying mechanisms show clear differences. The pristine cellulose hydrogel reduces free water as hydroxyl groups form extensive hydrogen bonds. The STA hydrogel further limits Zn^2+^ solvation as highly polarized metal‐oxygen bonds introduce abundant coordination sites and effectively disrupt the hydrogen‐bond network (Figure 3a,b).

To gain further mechanistic insight into the role of STA in regulating water structure and Zn^2+^ solvation, we conducted density functional theory (DFT) calculations to evaluate the interactions among key components in the STA hydrogel. The results show that the W═O terminal bonds on the STA surface primarily interact with the hydrogen atoms of nearby water molecules through charge redistribution, forming hydrogen bond‐like attractions (Figure 3f). The calculated adsorption energy of STA‐H_2_O interactions is significantly higher than that between water molecules themselves, suggesting that STA can effectively disrupt the native hydrogen‐bonding network of water. Moreover, STA exhibits a stronger binding affinity for Zn^2+^ compared to water, with adsorption energies exceeding the Zn^2+^ hydration energy. This indicates that STA can partially replace water molecules in the first solvation shell of Zn^2^⁺, facilitating desolvation. By promoting preferential water molecules rearrangement and competing effectively for Zn^2+^ coordination, these interactions synergistically restructure the local electrolyte environment and significantly reduce the Zn^2+^ solvation degree in the STA hydrogel relative to conventional ZnSO_4_ aqueous systems.

To experimentally validate the modulation of water structure and Zn^2^⁺ solvation in the STA hydrogel, we further analyzed the hydrogen‐bonding environment using the Fourier transform infrared (FTIR) spectroscopy. The O─H stretching vibration bands of water molecules shift depending on the surrounding hydrogen bonding strength as shown in Figure S17 (Supporting Information). In liquid electrolyte, Zn^2+^─H_2_O coordination induces local polarization, strengthening inter‐water hydrogen bonds and leading to a redshift in hydroxyl stretching band relative to that of pure water. However, in the pristine cellulose hydrogel, strong hydrogen bonding between water and cellulose increases environmental rigidity, resulting in a blueshift of the hydroxyl stretching band. Upon STA incorporation, the hydrogen bonding network is partially disrupted and reorganized via strong electrostatic interactions, forming a less hydrogen‐bonded but more dynamically active water structure, further shifting the hydroxyl vibration to higher frequencies. This restructured free water environment in the STA hydrogel is further confirmed by differential ATR‐FTIR spectra. As shown in Figure 3g, the STA system displays a marked decrease in signals from strongly hydrogen‐bonded O─H groups and a relative increase in weakly bonded or free water species. These spectral changes clearly reflect a disrupted hydrogen‐bond network and support the formation of a less coordinated and more dynamic water structure.

These structural characteristics of hydration can also be experimentally verified by the results of Raman spectroscopy (Figure 3h). The O─H stretching region of the spectra was deconvoluted into distinct sub‐bands corresponding to different hydrogen‐bonding states, including strongly hydrogen‐bonded, weakly hydrogen‐bonded, and free water species. By quantitatively analyzing the peak area ratios (Figure 3i), we found that the STA hydrogel contains a significantly lower proportion of strongly hydrogen‐bonded water and a higher proportion of weakly bonded and free water compared to other electrolytes. This indicates a substantial disruption of the extended hydrogen‐bond network, reflecting STA's ability to reorganize local water structures. In addition, ^1^H nuclear magnetic resonance (NMR) spectroscopy further supports these findings (Figure 3j). Compared to the water in liquid electrolyte, the water in pristine cellulose hydrogel displays broadened proton signals with a downfield shift, indicative of dense hydrogen bonding and heterogeneous water environments.^[^ ^28^, ^29^, ^30^ ^]^ In STA‐crosslinked hydrogel, water proton peaks remain broadened but shift slightly up‐field, suggesting weakened hydrogen bonding due to STA‐induced reorganization of the local structure. This restructuring is substantially beneficial to promote the formation of directional water channels, lowers the energetic barrier for Zn^2+^ diffusion, and facilitates more efficient Zn^2+^ transport crossing the hydrogel‐electrode interface.

To substantiate the spectroscopic evidence regarding water coordination environments, we conducted electrochemical measurements to assess the interfacial double‐layer capacitance of various electrolyte systems (Figure S18, Supporting Information). This technique offers a quantitative indicator of water binding strength at the electrode‐electrolyte interface. A lower interfacial capacitance corresponds to reduced dielectric polarization, typically associated with weaker water coordination. Among all tested systems, the STA hydrogel electrolyte exhibited the lowest interfacial capacitance, indicating a minimal degree of water binding. These findings are consistent with the differential ATR‐FTIR and Raman spectroscopy results, collectively confirming that STA incorporation disrupts the extensive hydrogen‐bonding network and facilitates the formation of a weaker hydrogen‐bonding aqueous environment.

**Figure 4:**
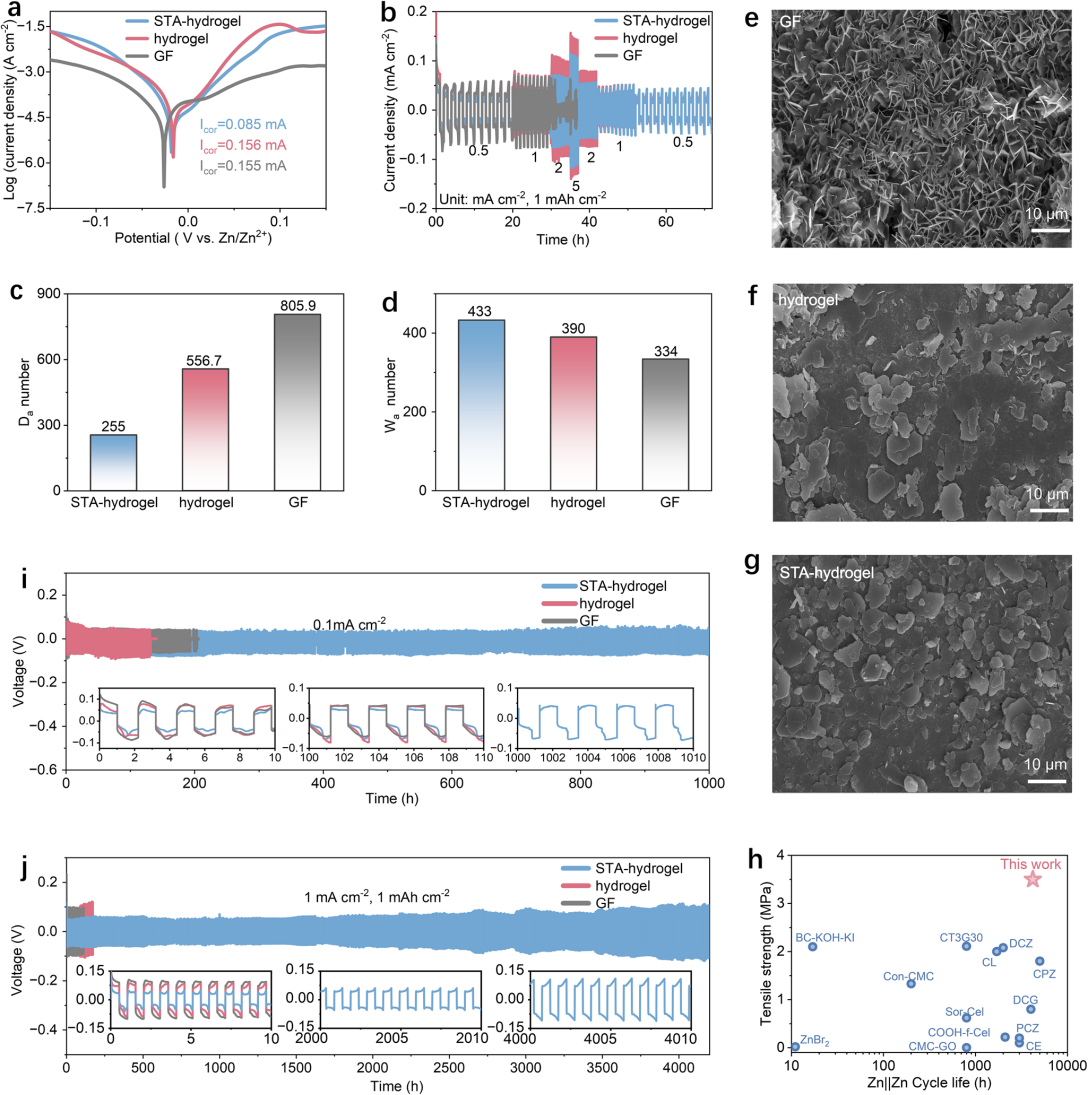
Electrochemical strapping‐plating evaluation of STA hydrogel electrolytes. a) Tafel curves of STA hydrogel electrolyte, pristine hydrogel electrolyte, and liquid electrolyte. b) Rate performance of Zn||Zn symmetric cells with three electrolytes in varied current density with a capacity of 1 mAh cm^−2^. c) *Da* number. d) *Wa* number. SEM images of Zn anode after 50 cycles (at 1 mA cm^−2^) in Zn||Zn symmetric cells with e) liquid electrolyte, f) pristine hydrogel electrolyte, and g) STA hydrogel electrolyte. h) Comparison of the mechanical strength and symmetric Zn||Zn cell cycling stability. Galvanostatic cycling performance of Zn||Zn symmetric cells at i) 1 mA cm^−2^ and 1 mAh cm^−2^, j) 0.1 mA cm^−2^ and 0.1 mAh cm^−2^.

Furthermore, we systematically studied electrochemical strapping/plating behavior of the STA hydrogel electrolyte. Tafel curve measurements indicated that the STA hydrogel electrolyte reduces the corrosion current and overpotential of zinc metal electrodes compared to pristine cellulose hydrogels and liquid electrolytes (**Figure** **4**a). This suggests that STA hydrogel effectively suppresses zinc electrode corrosion, improving the efficiency of zinc redox reactions and enhancing the cycle stability and reversibility of the battery. Additionally, we compared the cycling performance of Zn─Zn symmetric cells with different electrolytes under increasing current densities. The results showed that zinc electrodes paired with STA hydrogel electrolyte exhibited minimal voltage fluctuations and no short‐circuiting, demonstrating superior electrochemical reversibility and cycle stability (Figure 4b).

In addition, the growth of zinc dendrites during the electrochemical strapping/plating process plays a critical role in cycle stability. We quantitatively studied the relative control mechanisms of zinc deposition by collecting the *Damköhler* (*Da*) and *Wagner* (*Wa*) numbers of different electrolytes.^[^ ^31^, ^32^, ^33^ ^]^ The results reveal that STA hydrogel electrolyte exhibits a smaller Da number (Figure 4c) but a larger Wa number (Figure 4d; Figure S19 and Table S3, Supporting Information) compared to that of pristine cellulose hydrogels and liquid electrolytes. This indicates that although Zn^2+^ transport is faster within the STA hydrogel electrolyte, the interfacial reaction is slower. The high Wa number enhances the electric field‐driven migration of Zn^2+^, promoting a more uniform ion distribution. Meanwhile, the lower Da number suggests limited interfacial reactions, leading to fewer uneven deposition sites on the Zn electrode surface. The combined effect of strong electric field‐driven migration and stable hydrated channels mitigates concentration polarization and uneven interfacial reactions during Zn deposition. Moreover, the more stable Zn^2+^ transport channels in the STA hydrogel electrolyte likewise facilitate the formation of a more uniform deposition layer on the electrode surface. This mechanism can be further supported by the results of scanning electron microscope (SEM) images (Figure 4e–g; Figure S20, Supporting Information) and X‐ray diffraction (XRD) profiles (Figure S21, Supporting Information), where the planar deposition morphology of Zn crystals when paired with the STA hydrogel electrolyte obviously contrasts with the vertical or random growth of zinc dendrites in the liquid electrolyte.

We evaluated the cycling performance of different electrolytes in Zn─Zn symmetric cells. It has been generally recognized that hydrogel electrolytes tend to perform poor reversibility under low current densities due to weak electric field strength and sluggish ion migration, leading to uneven deposition and interface passivation. However, under a very low current density of 0.1 mA cm^−2^, Zn─Zn cells paired with STA hydrogel were able to sustain stable cycling for over 1000 h, far exceeding the performance of pristine cellulose hydrogel (≈200 h) and liquid electrolytes (≈150 h), although some asymmetry stripping/plating was observed at later stages (Figure 4i). It should be noted that slight fluctuations are observed in the charge–discharge curves during long‐term cycling at this relatively low current density, which is a common feature in hydrogel‐based electrolytes. Unlike the relatively smooth profiles obtained under high‐rate conditions, small current densities lead to slower ion migration and limited electrochemical deposition kinetics, resulting in dynamic voltage behavior. This phenomenon reflects the inherently lower ionic conductivity and more complex transport environment of hydrogel systems, where Zn^2^⁺ movement and interfacial processes tend to be more sensitive to minor perturbations. Such behavior has also been frequently observed in recently reported hydrogel‐based quasi‐solid‐state electrolytes, highlighting its generality across similar systems.

At a standard current density of 1 mA cm^−2^ (1 mAh cm^−2^), Zn─Zn cells with STA hydrogel can operate over 3000 h (1500 cycles) of stable cycling, while those using pristine cellulose hydrogels and liquid electrolyte show short‐circuit failure after ≈200 and ≈100 h, respectively (Figure 4j). Throughout this test, the charge–discharge profiles of Zn─Zn cells with STA hydrogel remained highly stable without barely obvious potential fluctuations. To eliminate device‐specific deviation, we also tested multiple Zn─Zn cells paired with the STA hydrogel (Figure S22, Supporting Information). All tested cells showed good cycling stability exceeding 3000 h at 1 mA cm^−2^, and in some cases, stability extended beyond 4000 h (>2000 cycles). In addition, the Zn electrode exhibits significantly improved cycling stability in the test of deep stripping (DOD = 50%) when paired with the STA hydrogel electrolyte, outperforming both the pristine cellulose hydrogel and the liquid electrolyte counterparts (Figure S23, Supporting Information). These results clearly demonstrate the ability of the STA hydrogel to enhance the electrochemical reversibility of Zn electrodes.

To better highlight the overall performance of the cellulose hydrogel electrolyte reported in this work, we conducted a comprehensive literature survey and added a comparative figure (Figure 4h; Table S4, Supporting Information) to benchmark its properties against previously reported systems. During our survey, we found that literature specifically focused on pure cellulose hydrogel electrolytes is extremely limited. Therefore, we extended our scope to include cellulose‐based composite hydrogel electrolytes as well, and systematically collected and summarized their electrochemical cycling lifespans and mechanical performances. As clearly shown in Figure 4h, the STA hydrogel electrolyte demonstrates significantly superior electrochemical stability and mechanical robustness compared to both pure and composite cellulose hydrogel systems reported in the literature. Particularly noteworthy is that the STA hydrogel achieves a remarkable elongation at break of up to 80%, alongside a high toughness of 1.5 MJ m^−3^. To the best of our knowledge, such a combination of mechanical flexibility and toughness has not been reported previously for pure cellulose hydrogel electrolytes, further underscoring the uniqueness and potential of our system for flexible energy storage applications.

**Figure 5:**
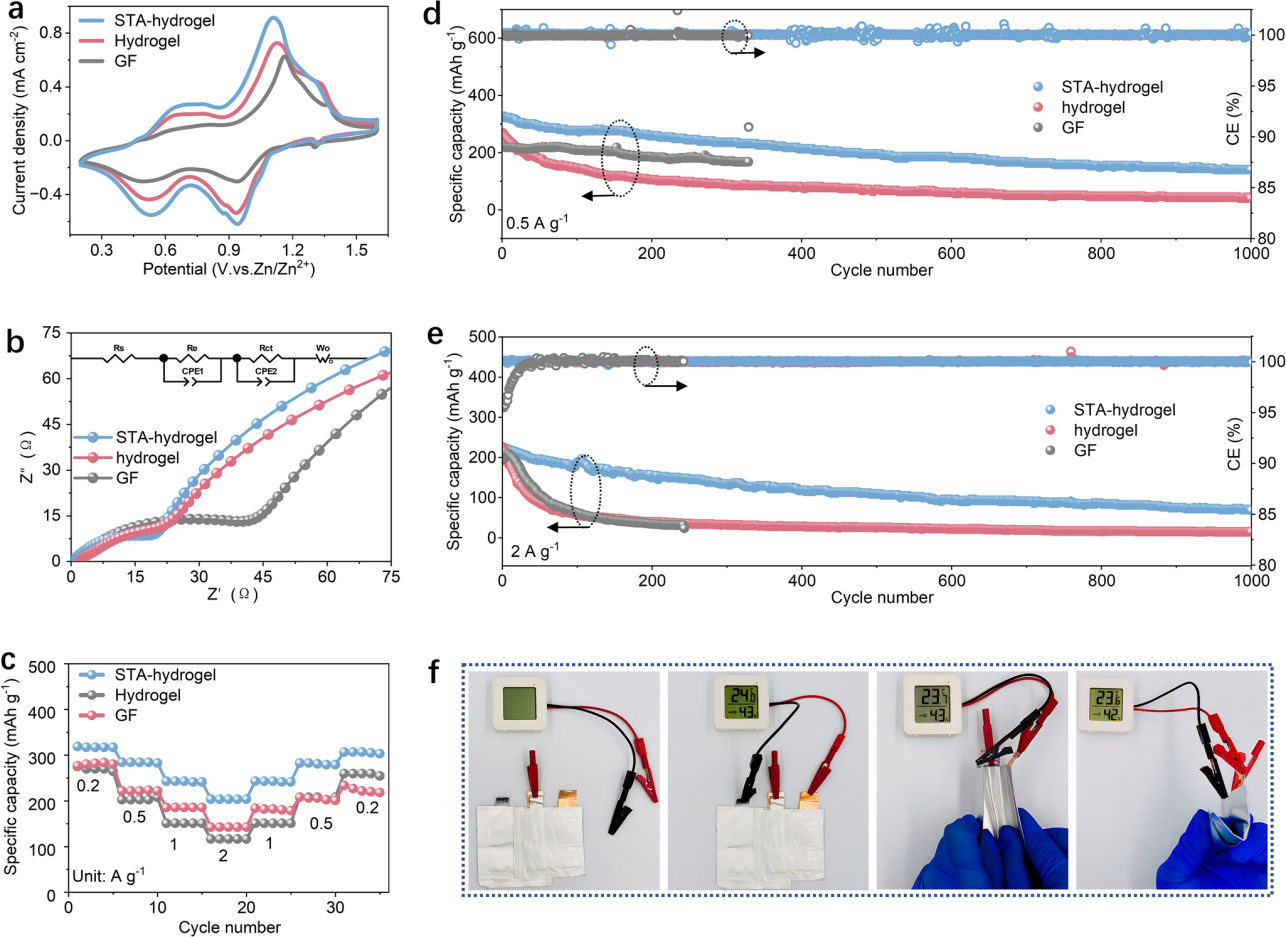
Full battery performance of STA hydrogel electrolytes by using V_2_O_5_ as cathode. a) CV curves of Zn||V_2_O_5_ full cells with three electrolytes at 1 mV s^−1^. b) EIS Nyquist plots of Zn||V_2_O_5_ full cells with three electrolytes. c) Rate performance of Zn||V_2_O_5_ full cells with three electrolytes. Long‐term cycling performance of Zn||V_2_O_5_ full cells with three electrolytes at d) 0.5 A g^−1^, e) 2 A g^−1^. f) Schematic illustration for the flexibility of the assembled pouch cell.

We further evaluated the performance of STA hydrogel electrolyte in the full battery device using V_2_O_5_ as the cathode material. In fact, thetransport dynamics of Zn^2+^ in the electrolyte significantly impact the chemical intercalation/extraction kinetics of Zn^2+^ and reversibility in the V_2_O_5_ crystal structure, thus determining the effective utilization of the active sites in the cathode and the overall capacity of the full battery. Additionally, the stability of the electrolyte also plays a crucial role in suppressing the dissolution and structural collapse of the V_2_O_5_ crystal, enhancing the structural integrity of the zinc storage reaction and improving cycling capacity retention. First, we collected the cyclic voltammetry (CV) curves of Zn‐V_2_O_5_ full batteries assembled with three different electrolytes (**Figure** **5**a). Two pairs of electrochemical peaks are clearly observed. The first pair of peaks at lower voltage corresponds to the initial insertion of Zn^2+^ into the V_2_O_5_ layered structure, accompanied by the reduction of V^5+^ to V^4+^, representing the initial high‐potential intercalation process.^[^ ^34^, ^35^ ^]^ The second pair of peaks at higher voltage represents further insertion of Zn^2+^, involving deeper intercalation or phase transition, with V potentially being further reduced to V^3+^. Compared to pristine cellulose hydrogels and liquid electrolytes, the voltage difference between the intercalation and extraction peaks in the CV curve of the STA hydrogel electrolyte is smaller, indicating that the STA hydrogel electrolyte reduces the polarization in the V_2_O_5_ cathode, thus optimizing the reaction kinetics and improving the efficiency of electron and ion transport. Moreover, the integrated peak area in the CV curve of the STA hydrogel is significantly larger than in the other two electrolytes, indicating a larger electrochemical capacity. Additionally, in the Nyquist plot of electrochemical impedance spectroscopy (EIS), the Zn‐V_2_O_5_ full battery with STA hydrogel electrolyte exhibits a smaller semicircle radius and steeper low‐frequency slope, reflecting better charge transfer and ion diffusion capabilities (Figure 5b). As a result, the Zn‐V_2_O_5_ full battery with STA hydrogel electrolyte shows a smaller capacity decay with increasing current density and a relatively higher specific capacity, demonstrating better rate performance compared to that of pristine cellulose hydrogels and liquid electrolytes (Figure 5c).

When cycling at a current density of 0.5 A g^−1^ (0.85C), the specific capacity of the STA hydrogel electrolyte‐based Zn‐V_2_O_5_ full battery remains above 200 mAh g^−1^ even after 1000 cycles, with a stable Coulombic efficiency of ≈100% (Figure 5d), significantly outperforming the pristine cellulose electrolyte (<100 mAh g^−1^). Its cycling stability is also much better than that of liquid electrolytes, which exhibit obvious short‐circuit failure after only 300 cycles. When the current density is increased to 2 A g^−1^ (3.4C), the specific capacity of the STA hydrogel electrolyte‐based Zn‐V_2_O_5_ full battery remains ≈100 mAh g^−1^ after 1000 cycles, demonstrating also well cycling stability (Figure 5e). In addition, the STA hydrogel electrolyte can obviously suppress self‐discharge in full batteries. After resting for 72 h at full charge, Zn‐V_2_O_5_ batteries with the STA hydrogel retain up to 85% of their initial capacity, significantly larger than those with pristine cellulose hydrogel (36%) and liquid electrolyte (58%) (Figure S24, Supporting Information). This improvement can be attributed to the unique water hydrogen‐bonding network in the STA hydrogel, which effectively mitigates parasitic reactions and enhances corrosion resistance. More remarkably, the Zn‐V_2_O_5_ full battery with STA hydrogel electrolyte maintains stable operation even under large mechanical deformations or severe bending, demonstrating excellent mechanical flexibility and electrochemical stability (Figure 5f; Video S1, Supporting Information).

## 3 Conclusion

In summary, we have developed a cellulose hydrogel electrolyte that combines high mechanical toughness with low Zn^2+^ solvation, which can effectively enhance both cycling stability and flexibility of AZIBs. This hydrogel is enabled by a novel chemical dissolution and crosslinking strategy that allows cellulose to dissolve efficiently in a neutral environment without molecular degradation. Meanwhile, the incorporation of the STA promotes strong hydrogen bonding, forming robust crosslinks between cellulose chains. The resulting hydrogel features high molecular weight and dense crosslinking, leading to excellent mechanical strength and toughness. More significantly, STA can reorganize the water hydrogen‐bonding network through strong metal‐oxygen polarization. This not only reduces the Zn^2+^ solvation but also establishes efficient ion transport pathways. As a result, the STA hydrogel exhibits a wide electrochemical stability window and high ionic conductivity that can effectively suppress parasitic reactions and facilitate uniform planar Zn deposition via balanced electrochemical transport‐reaction dynamics. Electrochemical testing shows stable cycling of Zn─Zn symmetric cells for over 3000 h. Full battery using V_2_O_5_ cathodes retains capacities above 200 mAh g^−1^ even after 1000 charge–discharge cycles. Notably, soft‐packed Zn‐V_2_O_5_ batteries assembled by the STA hydrogel maintain stable operation under mechanical deformation and bending, demonstrating exceptional flexibility and durability. As far as we know, this represents currently one of the few reports of a fully cellulose‐based hydrogel electrolyte enabling flexible AZIB without additional polymer additives. This work provides a green, low‐cost, and scalable strategy for next‐generation flexible AZIBs through hydrogel electrolyte design.

## Conflict of Interest

The authors declare no conflicts of interest.

## Supporting information



Supporting Information

Supplemental Video 1

## Data Availability

The data that support the findings of this study are available from the corresponding author upon reasonable request.
