# Supplementary material for: Tough Cellulose Hydrogel Electrolyte with Low Solvation for Highly Reversible and Flexible Aqueous Zinc‐Ion Battery
Source: Adv Sci (Weinh). 2025 Jul 29;12(40):e11759. doi: 10.1002/advs.202511759 (PMC12561410; doi:10.1002/advs.202511759)
Supplement: Supplementary file 1 — Supporting Information [file ADVS-12-e11759-s001.docx]

**Supporting Information**

**Tough Cellulose Hydrogel Electrolyte with Low Solvation for Highly Reversible and Flexible Aqueous Zinc-Ion Battery**

*Fan Chen, Xuan Li, Shi-Peng Chen, Yilin Zhang, Hua-Dong Huang, Hongli Yang*, Shengyang Zhou*, Zhong-Ming Li*

*Fan Chen, Xuan Li, Yilin Zhang, Shengyang Zhou*

College of Materials Science and Engineering, Sichuan University, Chengdu 610065, China

*Shi-Peng Chen, Hua-Dong Huang, Hongli Yang, Zhong-Ming Li*

College of Polymer Science and Engineering, Sichuan University, Chengdu 610065, China

*Zhong-Ming Li*

West China Hospital/West China School of Medicine, Sichuan University, Chengdu 610041, China

*Hua-Dong Huang, Hongli Yang, Shengyang Zhou, Zhong-Ming Li*

National Key Laboratory of Advanced Polymer Materials, Sichuan University, Chengdu 610065, China

*^*^*E-mail: [shengyang.zhou@scu.edu.cn](mailto:shengyang.zhou@scu.edu.cn); [yang.hongli@scu.edu.cn](mailto:yang.hongli@scu.edu.cn)

**Experimental Section**

**Materials.** Zinc sulfate heptahydrate (ZnSO_4_·7H_2_O, AR, Aladdin), zinc foil (50 μm, Olegeeino), cooper foil (50 μm, Olegeeino), V_2_O_5_ foil (Future Materials Tech. Jilin. Co., Ltd), glass fiber (Olegeeino), silicotungstic acid hydrate (H_4_O_40_SiW_4_·xH_2_O, AR, Aladdin), 1,1,3,3-Tetramethylguanidine (TMG, 99%, Aladdin), Methoxyacetlc acid (MAA, 99%, Aladdin) and filter paper (BKMAM Holding Co., Ltd) were used in this study without any further purification. And ultra-pure water, with the resistivity of 18.2 MΩ · cm, was produced by an ultra-pure water system (Shanghai Lichen Instrument Technology Co., Ltd.).

**Preparation of STA-hydrogel and hydrogel solution.** Preparation of Cellulose solution. After dried at 60 °C for 24 h in advanced, filter paper was prepared as the source of cellulose. Then 56.07g TMAG and 43.93 g MAA, with the molar ratio of 5:5, were mixed by stirring for 5 min at room temperature to obtain transparent phase as the solvent. After the preparation of [TMG][MAA] solvent, 3g cellulose was added to 100g [TMG][MAA] solvent, and then stirred at a rate of 500 rmp at 80 °C to dissolve for 20 hours until cellulose fibrils and crystals could not be observed under polarized light microscopy (BX51, Olympus Co., Tokyo, Japan). The hydrogel solution was prepared by method above, while to obtain the STA-hydrogel solution, 0.1g silicotungstic acid hydrate (STA) was added to the solution above and continued to stir at same condition for 2 hours.

**Preparation of STA-hydrogel and hydrogel electrolytes.** First, the STA-hydrogel solution was put in oven at 80 ℃ for 12 hours to remove bubbles. Second, the solution was coated onto a glass substrate by using a Myer rod to obtain a uniform film, with the thickness of 200 μm. Then, the STA-hydrogel electrolyte was prepared by soaking the substrate in ultra-pura water for 24 hours then in 2 M ZnSO_4_ for 24 hours. The preparation of hydrogel electrolyte was the same as that of the STA-hydrogel electrolyte. Finally, both two electrolytes were cut into discs with a diameter of 16 mm to assemble button cells.

**Materials characterization.** The X-ray diffraction (XRD) patterns were obtained by an X-ray diffractometer with Cu K_α_ radiation (DX-2700B, HAOYUAN Instrument). CPD and Ambient pressure dried (APD), (Autosamdri-, Tousimis, USA), were used to pre-prepare samples which were used to carry out SEM and EDS. The scanning electron microscopy (SEM) images and the energy-dispersive X-ray spectroscopy (EDS) mapping images were collected by a Nova Nano-SEM (450, FEI). The Fourier transform infrared spectroscopy (FTIR) was measured by a spectroscopy (Nicolet 6700, Thermo-Fisher Scientific, USA) with the wavenumber from 700 to 4000 cm^-1^. The tensile tests were measured using a length of 10 cm at a strain rate of 5 mm min^-1^ on a testing machine (5699, Instron, USA). The Rama mapping and point spectrum were obtained by a Laser micro-Raman imaging spectrometer (DXIS, USA) with the wavelength of 532 nm. The X-ray photoelectron spectra (XPS) was recorded on a Axis ultra DLD. The proton nuclear magnetic resonance (NMR) was obtained by NMR (Avance 400 MHz, Bruker, Germany). The Thermogravimetric analysis (TGA) was carried out on a thermo-gravimetric analyzer (TG209F1, Netzsch Scientific, Germany) in the range of 30 °C–800 °C at a heating rate of 10 °C min^−1^ under a nitrogen flow. The 2D-Small-angle X-ray scattering (SAXS) patterns were recorded using an X-ray CCD detector of PILATUS3 2M with a pixel array of 1475 × 1679 pixels at the beamline BL10U1 (λ = 1.24 Å), Shanghai Synchrotron Radiation Facility (SSRF, Shanghai, China). The detector has a pixel size of 172 × 172 μm^2^ and the distance between the sample and detector was 5905.4 mm. The 1D-SAXS intensity for each scattering vector *q* were obtained by integrating over the azimuthal range of 0-360^o^ from the 2D pattern.

**Modeling of SAXS data.** A generalized empirical two-region Guinier-Porod model (proposed by Hammouda[^1^](#_ENREF_1)) with Guinier and Porod regions was used to fit the SAXS data. It describes the form factor for nonspherical objects with the following Equation (1):

$$\begin{aligned} I\left( q \right)=\left\{ \begin{aligned} \frac{G}{q^{s}}\mathrm{ex}p \left( \frac{-q^{2}R_{g}^{2}}{3-s} \right)I\left( q \right)=\frac{G}{q^{s}}\exp(\frac{-q^{2}R_{g}^{2}}{3-s}) , q\leq q1 \\ I\left( q \right)=\frac{D}{q^{\alpha}} , q\geq q1 \end{aligned} \right.\#\left( 1 \right) \end{aligned}$$

where *q* is the scattering vector, *I(q)* is the scattering intensity, *s* is the “dimensionality” parameters, and *Rg* is the characteristic length for the size of the scattering object, *α* is the Porod exponent, and *G* and *D* are the Guinier and Porod scale factors, respectively.

With the requirement for the constraint of continuity of the intensity function, its double derivative yields a transition region at *q_1_* and the following relationships are obtained, Equation (2):

$$\begin{aligned} \left\{ \begin{aligned} D=Gexp\left( \frac{-q_{1}^{2}R_{g}^{2}}{3-s} \right)q_{1}^{\left( \alpha-s \right)} \\ q_{1}=\frac{1}{R_{g}}\left[ \frac{\left( \alpha-s \right)\left( 3-s \right)\left( \alpha-s \right)\left( 3-s \right)}{2} \right]^{1/2} \end{aligned} \right.\#\left( 2 \right) \end{aligned}$$

**Electrochemical Characterization.** All the commercial Zn foil (50 μm), Cu foil (50 μm) and V_2_O_5_ foil were cut into discs with a diameter of 12 mm. Glass fiber was cut into discs with a diameter of 16 mm to add 80 μL 2M ZnSO_4_ electrolyte as a control experiment. And Zn-Zn symmetric cells, Zn-Cu asymmetric cells and Zn- V_2_O_5_ full cells with different electrolytes were assembled by using CR2032 coin-type cells. The battery charge-discharge tests were implemented using a Neware battery system (CT-4008T). And the other electrochemical measurements, including cyclic voltammetry (CV), chronoamperometry (CA), linear sweep voltammetry (LSV), electrochemical impedance spectroscopy (EIS) and Tafel plots, electrochemical stable window (ESW) were conducted by using an electrochemical workstation (CHI660E, Chenhua). EIS tests conducted over the range of 10^-1^ to 10^5^ Hz. Tafel tests were performed between -0.2 V and 0.2 V under the scan rate of 1 mV s^-1^. CV tests for Zn||Cu asymmetric cells were carried out between -0.2 V and 0.5 V at 1 mV s^-1^. CV tests for Zn||V_2_O_5_ full cells were carried out between 0.2 V and 1.6 V at 1 mV s^-1^.

**Zn^2+^ Diffusion Coefficient Measurements.** The diffusion coefficients of Zn^2+^ (D_Zn_^2+^) were measured using Zn||V_2_O_5_ full cells by Galvanostatic Intermittent Titration Technique (GITT). The GITT tests were performed with a charge-discharge pulse of 15 s at 0.5 A g^-1^ and a rest period of 900 s. And the diffusion coefficients of Zn^2+^ (D_Zn_^2+^) were calculated by Equation (3):

$$\begin{aligned} D=\frac{4}{\pi\tau}\left( \frac{n_{m}V_{m}}{S} \right)^{2}\left( \frac{\Delta E_{s}}{\Delta E_{t}} \right)\#\left( 3 \right) \end{aligned}$$

Where *τ* is the duration of the current pulse (s), *n_m_* is the number of moles (mol), *V_m_* is the molar volume of the electrode (cm^3^ mol^-1^), *S* is the electrode area (cm^2^), *∆E_s_* is the steady-state voltage change, due to the current pulse and *∆E_t_* is the voltage change during the constant current pulse, eliminating the iR drop.

**Electrical Double Layer (EDL) measurements.** The capacitance is determined by the liner relationship between capacitance current (*i_c_*) and scan rate (*v*), which can be obtained from the slop of CV curves. So the C can be calculated by the following equation (4):

$$\begin{aligned} C=\frac{i_{c}}{v}\#\left( 4 \right) \end{aligned}$$

Where *i_c_=(i_0+_-i_0-_)/*2, meaning the half value of current difference during positive and negative scan at 0 V.

**Measurements of *D_a_* and *W_a_* number.** *Damköhler* number (*D_a_*) can be calculated by the following equation (5):

$$\begin{aligned} D_{a}=\frac{i_{0}}{i_{l}}\#\left( 5 \right) \end{aligned}$$

Where *i_0_* is the exchange current density which can be obtained from Tafel curves and *i_l_* is the diffusion-limiting current density, which can be calculated by Equation (6), as follows:

$$\begin{aligned} i_{l}=\frac{4FCD_{+}}{L}\#\left( 6 \right) \end{aligned}$$

Where *F* is Faraday constant, *C* is the salt concentration in the electrolyte, D_+_ is the cation diffusion coefficient, and L is the space between two separated electrodes.

*Wagner* number (*W_a_*) can be obtained by the following equation (7):

$$\begin{aligned} W_{a}=\frac{R_{\mathrm{ct}}}{R_{Ω}}\#\left( 7 \right) \end{aligned}$$

*R_ct_* is the charge transfer resistance from the EIS spectra and *R_Ω_* electrolyte resistance from the EIS spectra.

**Ionic Conductivity Measurements.** The ionic conductivities (*σ*) of the separators were measured using two stainless-steel electrodes. The *σ* can be calculated through the following equation (8):

$$\begin{aligned} \sigma=\frac{L}{RS}\#\left( 8 \right) \end{aligned}$$

where *L* is the thickness of the separator, 𝑅 is the resistance based on the EIS test, and *S* is the contact area between the separator and stainless-steel electrode.

**Zn^2+^ Transfer Number Measurements****.** Zn^2+^ transfer numbers (*t_Zn_^2+^*) of different separators were measured by assembling Zn||Zn symmetric cells based on the following calculation equation (9):

$$\begin{aligned} t_{{Zn}^{2+}}=\frac{I_{S}\left( \Delta V-I_{0}R_{0} \right)}{I_{0}\left( \Delta V-I_{S}R_{S} \right)}\#\left( 9 \right) \end{aligned}$$

where *∆𝑉* is the constant polarization voltage (40 mV), *I_0_* and *I_S_* are the initial current and the steady-state current based on CA test, respectively. *R_0_* and *R_s_* are the initial resistance and the stable resistance based on EIS test, respectively.

**Desolvation Activation Energy Measurements.** The desolvation activation energy (*E_a_*) of hydrated Zn^2+^ was measured using Zn||Zn cells at different temperatures. The *E_a_* can be calculated through the following equation (10):

$$\begin{aligned} \frac{1}{R_{ct}}=\mathrm{Aexp}\left( \frac{-E_{a}}{RT} \right)\frac{1}{R_{ct}}=\mathrm{Aexp}\left( \frac{-E_{a}}{RT} \right)\#\left( 10 \right) \end{aligned}$$

where *R_ct_* is the resistance obtained from the EIS spectra, *R* is the gas constant, and *T* is the thermodynamic temperature.

**MD simulations**. All simulations were performed using Materials Studio 2020.

Model Construction. Model hydrogel electrolyte: This model comprises eight cellulose chains (degree of polymerization, n = 5) and 892 water molecules. The structure belongs to the P1 space group within a triclinic crystal system, with lattice parameters of a = 34.01 Å, b = 34.84 Å, c = 33.57 Å, and angles α = 88.06°, β = 88.79°, and γ = 90.47°. Model STA-hydrogel electrolyte: This model consists of one silicotungstic acid hydrate molecule centrally positioned within the simulation box, eight cellulose chains (n = 5), and 892 water molecules. It also crystallizes in the P1 space group (triclinic system) with lattice parameters of a = 34.57 Å, b = 35.01 Å, c = 35.12 Å, and angles α = 88.27°, β = 88.27°, and γ = 89.18°. Simulation Protocol: Geometry optimizations and molecular dynamics (MD) simulations were subsequently performed using the forcite module. The energy convergence criterion was set to 2.0 × 10^-5^kcal/mol and the force convergence threshold to 0.001 kcal/mol/Å. The Universal Force Field (UFF) was employed for all simulations, with atomic charges assigned using the charge equilibration (QEq) method. Electrostatic and van der Waals interactions were computed using the ewald summation technique. MD simulations were conducted under NPT ensemble conditions at 298.15 K and 1 atm. Temperature was controlled using a Nosé–Hoover thermostat (damping parameter = 0.1 ps), and pressure was maintained using a Berendsen barostat (time constant = 1.0 ps). The system was equilibrated for 10 ns to ensure stability.

**DFT calculation**. All calculations were implemented in Materials Studio with the DMol3 code. The Perdew-Burke-Ernzerhof (PBE) functional of the generalized gradient approximation (GGA) was used to calculate the exchange-correlation energy. The DFT semi-core pseudopotentials (DSPPs) method was used to introduce a certain degree of relativistic correction to the atoms and the double numerical plus polarization (DNP) was chosen during the geometry optimization. The convergence tolerances of energy change, maximum force, and maximum displacement were set as 2 × 10^-5^ Ha, 0.004 Ha/Å, and 0.005 Å, respectively. The adsorption energy (Eads) of species is calculated by Equation (11):

$$\begin{aligned} E_{ads}=E_{sys}{- E}_{cata}-E_{spec}\#(11) \end{aligned}$$

where *E_sys_, E_cata_, and E_spec_* are the total energy of the optimized system with adsorbed species, the isolated catalyst, and species, respectively.

**Finite Element Analysis Simulation.** The multi-physics model of the electric field and zinc ion concentration field was simulated using COMSOL Multiphysics 6.2. The analysis area encompassed a 50 μm × 50 μm electrolyte area. 2 M ZnSO_4_ aqueous solution was set as the electrolyte and a voltage of 1.0 V was applied. The ion concentration diffusion obeys Fick’s laws. The ionic conductivity of GF, STA hydrogel and pristine hydrogel were 6.5 mS cm^-1^, 22.8 mS cm^-1^ and 5.7 mS cm^-1^. Diffusion coefficients were measured by GITT curves.

**Supplementary Results**

**
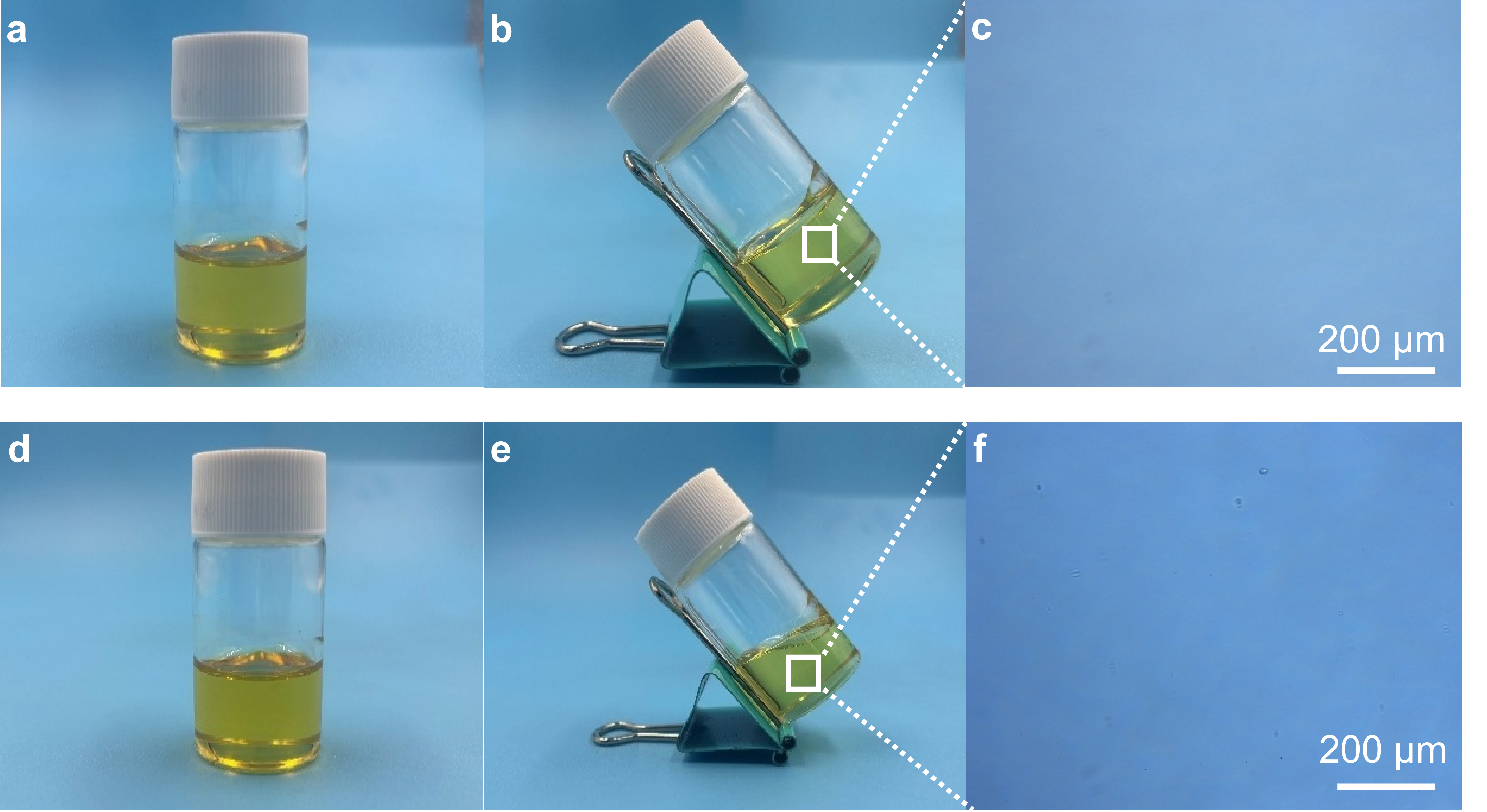
**

**Figure S1.** Photographs of (a), (b) STA hydrogel solution and (d), (e) pristine hydrogel solution. Polarizing microscope photographs at 10x magnification of (c) STA hydrogel solution, (f) pristine hydrogel solution.

**
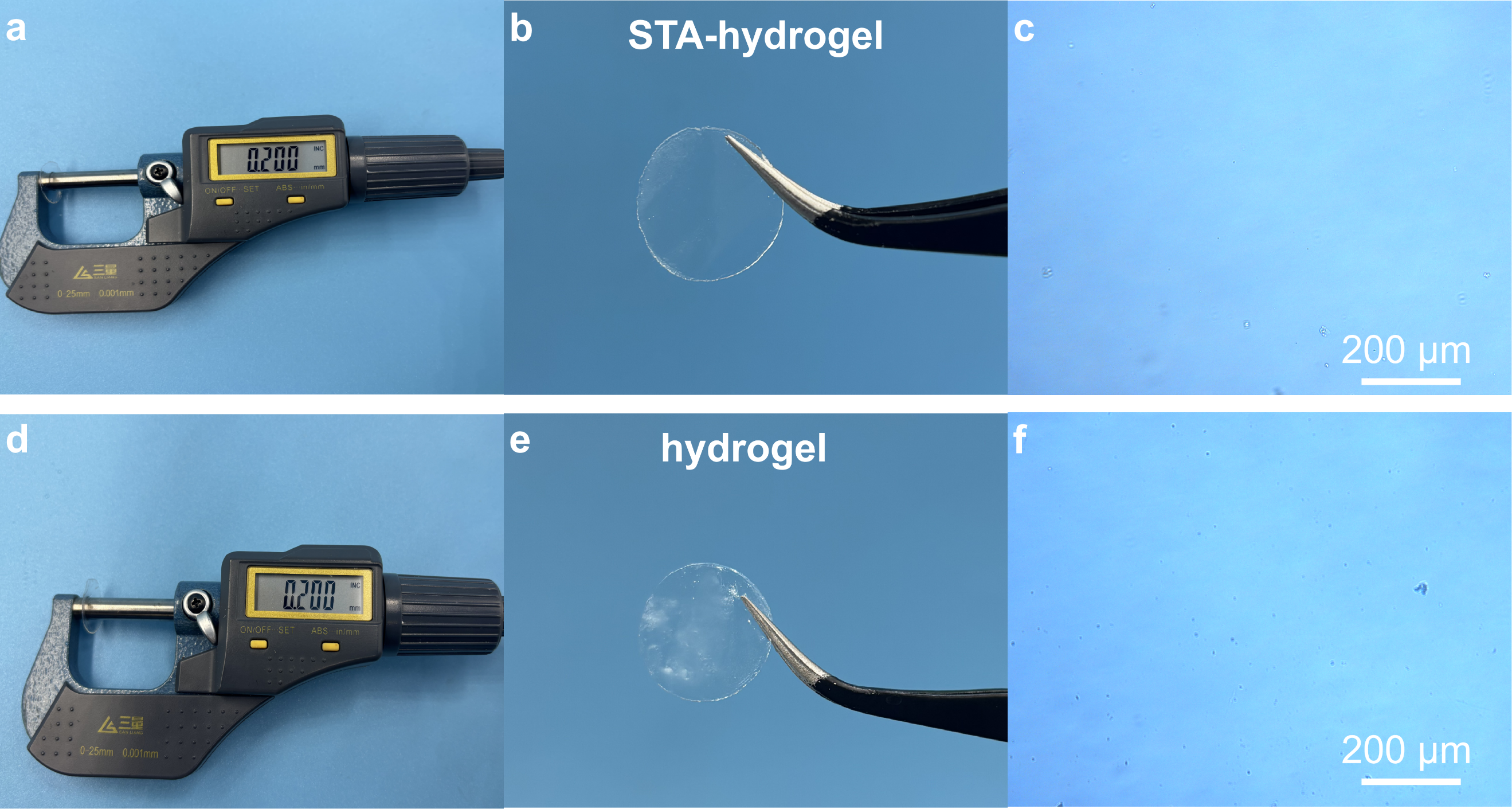
**

**Figure S2.** Thickness measurements of (a) STA hydrogel and (d) pristine hydrogel. (b) and (e) Photographs of cut-off electrolytes. Corresponding polarizing microscope photographs at 10x magnification of (c) STA hydrogel, (f) pristine hydrogel.


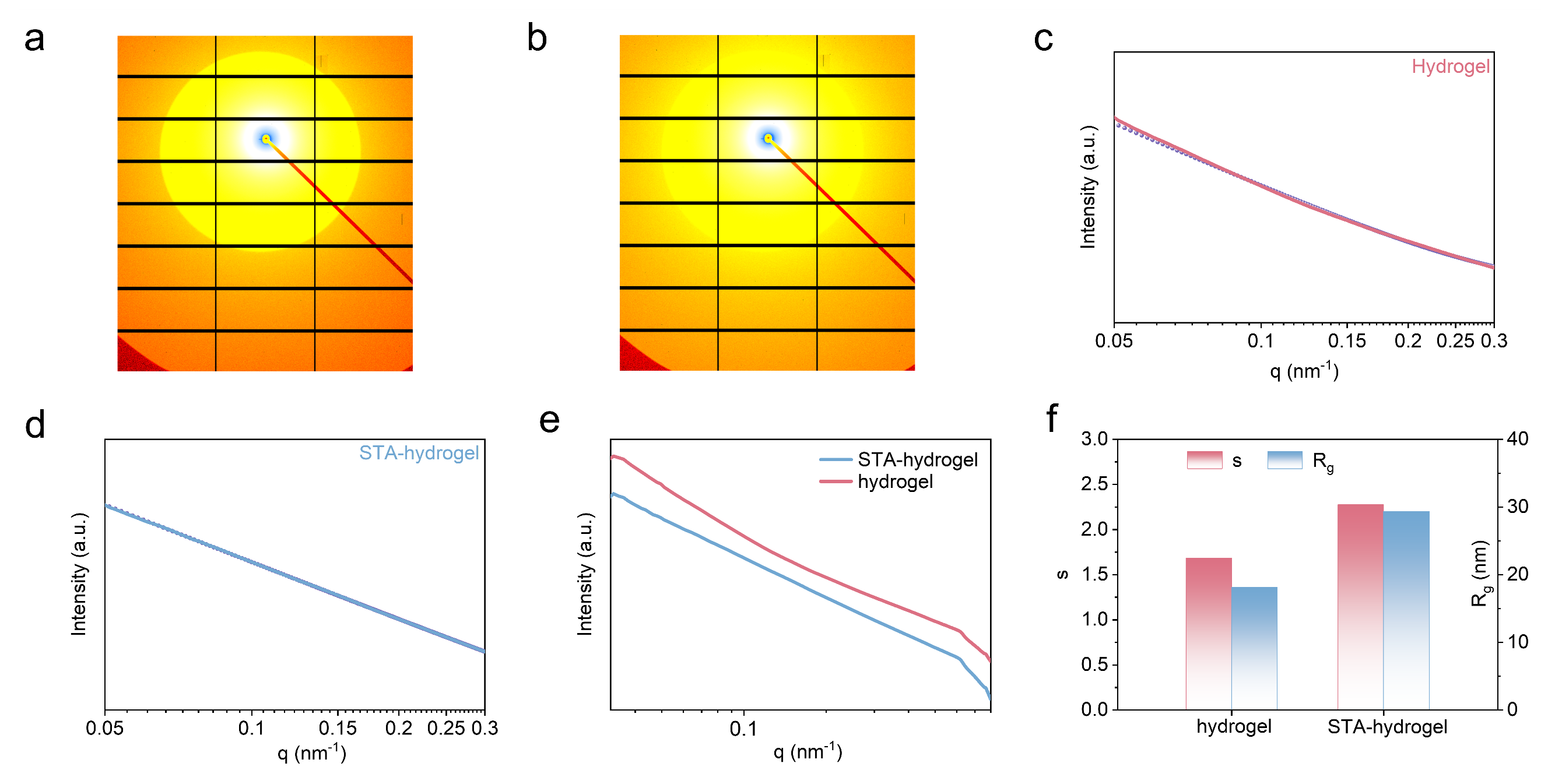


**Figure S3.** Representative 2D-SAXS patterns of (a) STA hydrogel and (b) pristine hydrogel. Corresponding 1D-SAXS intensity profiles and fitting curves of (c) pristine hydrogel, (d) STA hydrogel and (c) comparison profile. (f) Fitted parameters of Guinier-Porod model.


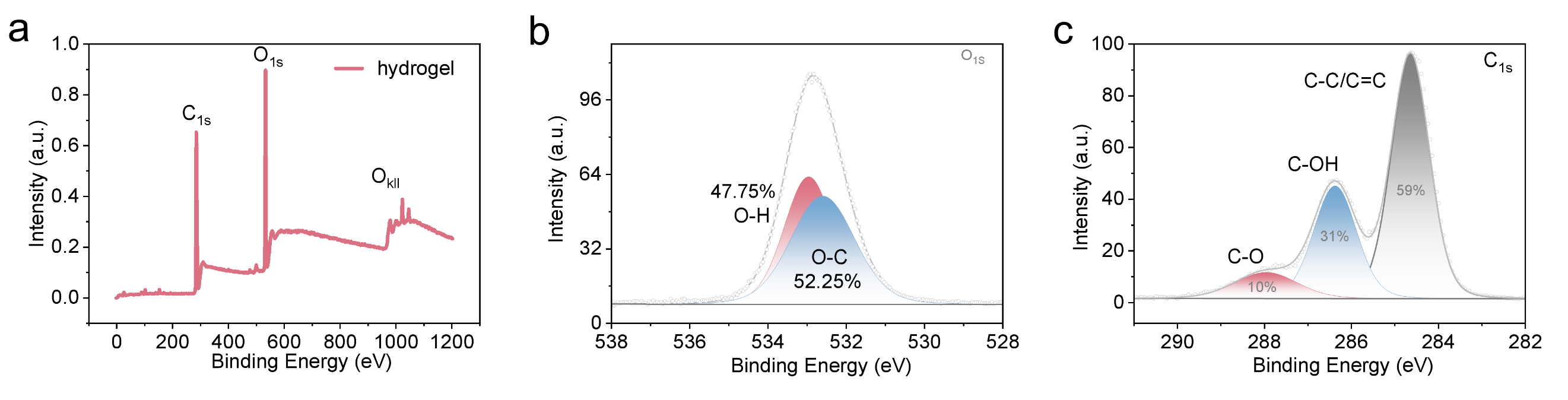


**Figure S4.** XPS spectra of pristine hydrogel. (a) the full XPS spectra. (b) corresponding peak fitting of O_1s_. (c) corresponding peak fitting of C_1s_.


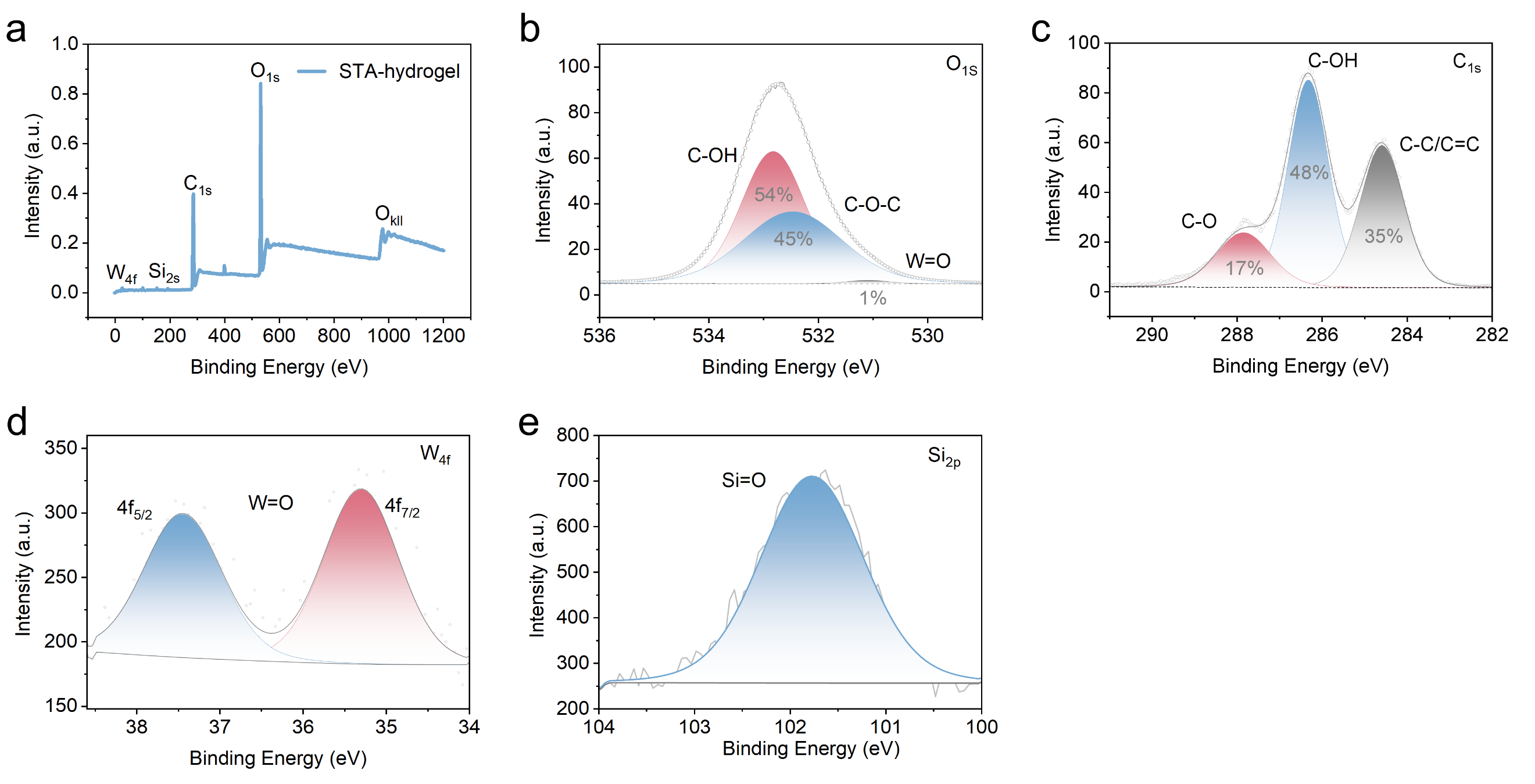


**Figure S5.** XPS spectra of STA hydrogel. (a) The full XPS spectra. (b) Corresponding peak fitting of O_1s_. (c) Corresponding peak fitting of C_1s_. (d) Corresponding peak fitting of W_4f_. (e) Corresponding peak fitting of Si_2p_.


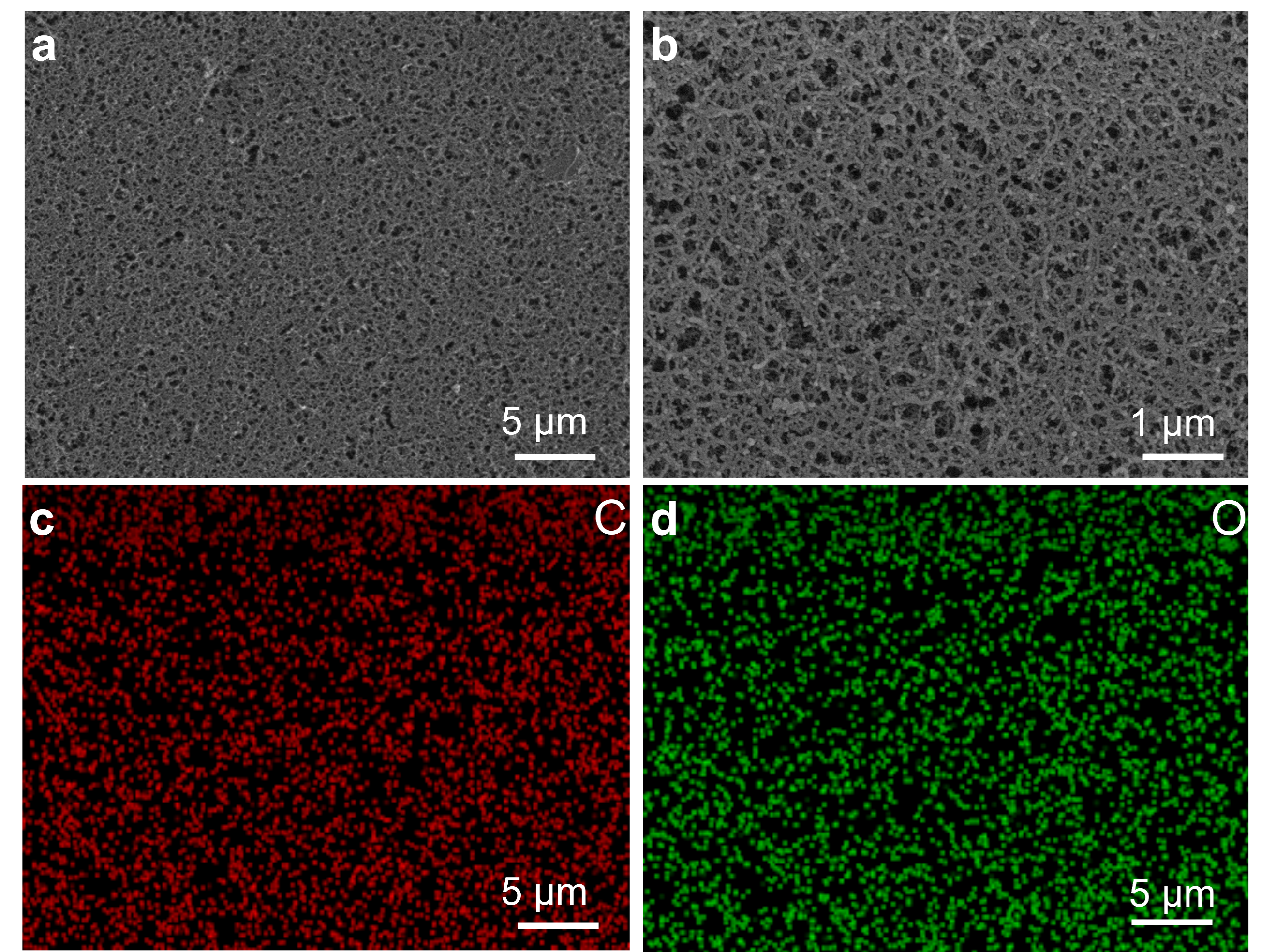


**Figure S6.** Surface FE-SEM images and corresponding elements EDS mapping images of pristine hydrogel prepared by CPD and APD in advance.

**Table S1.** Element distribution table of pristine hydrogel

| Element | At (%) |
| --- | --- |
| C | 57.39 |
| O | 42.61 |


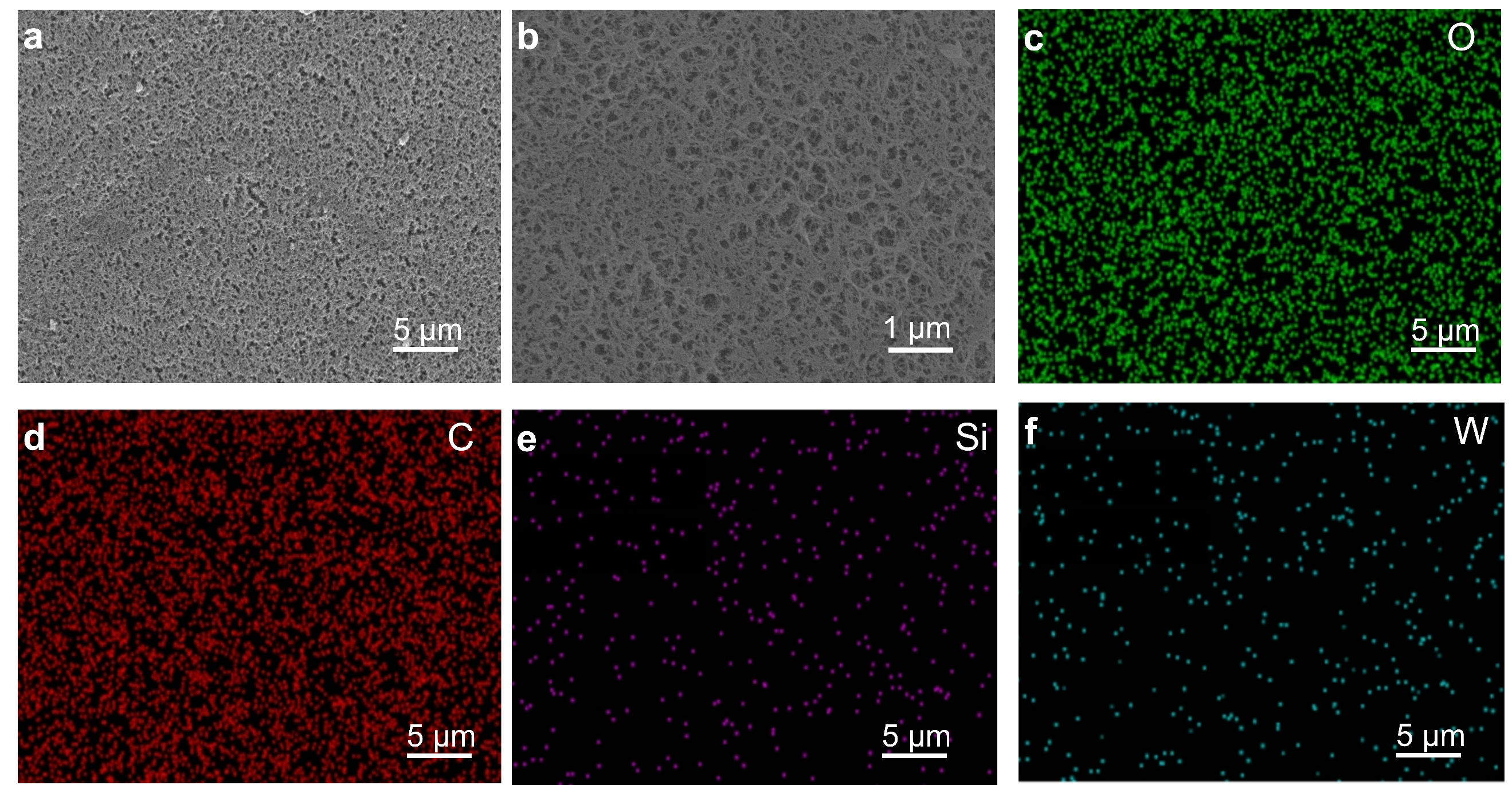


**Figure S7.** Surface FE-SEM images and corresponding elements EDS mapping images of STA hydrogel prepared by CPD and APD in advance.

**Table S2.** Element distribution table of STA hydrogel

| Element | At (%) |
| --- | --- |
| C | 58.77 |
| O | 41.07 |
| Si | 0.11 |
| W | 0.05 |


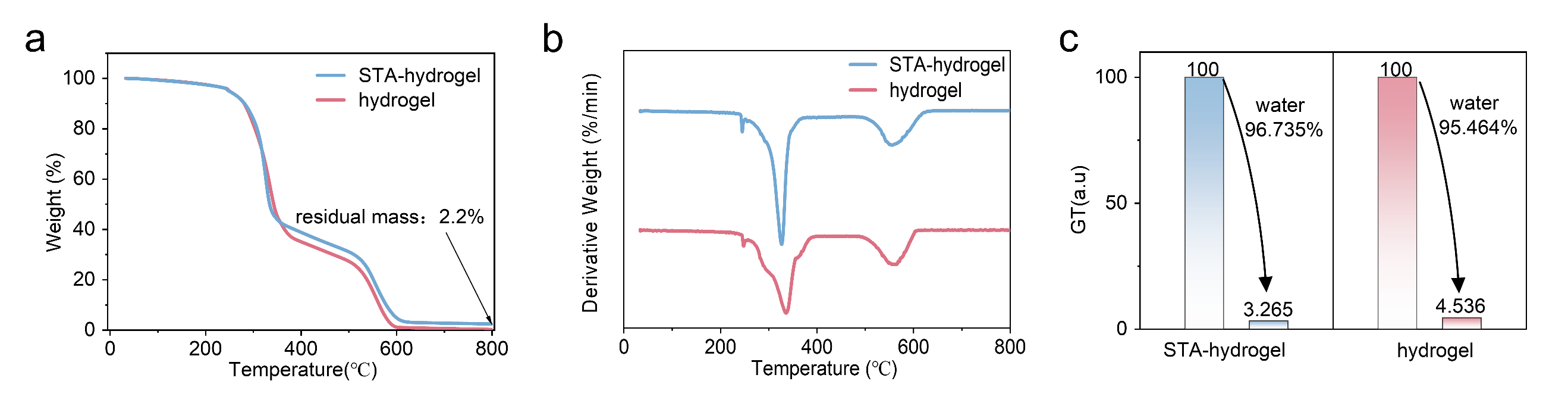
**Figure S8.** (a) TGA profiles of two dried hydrogels. (b) corresponding DGT thermograms. (c) Water content analysis, calculated by mass difference before and after drying at 80 °C for 12 hours.


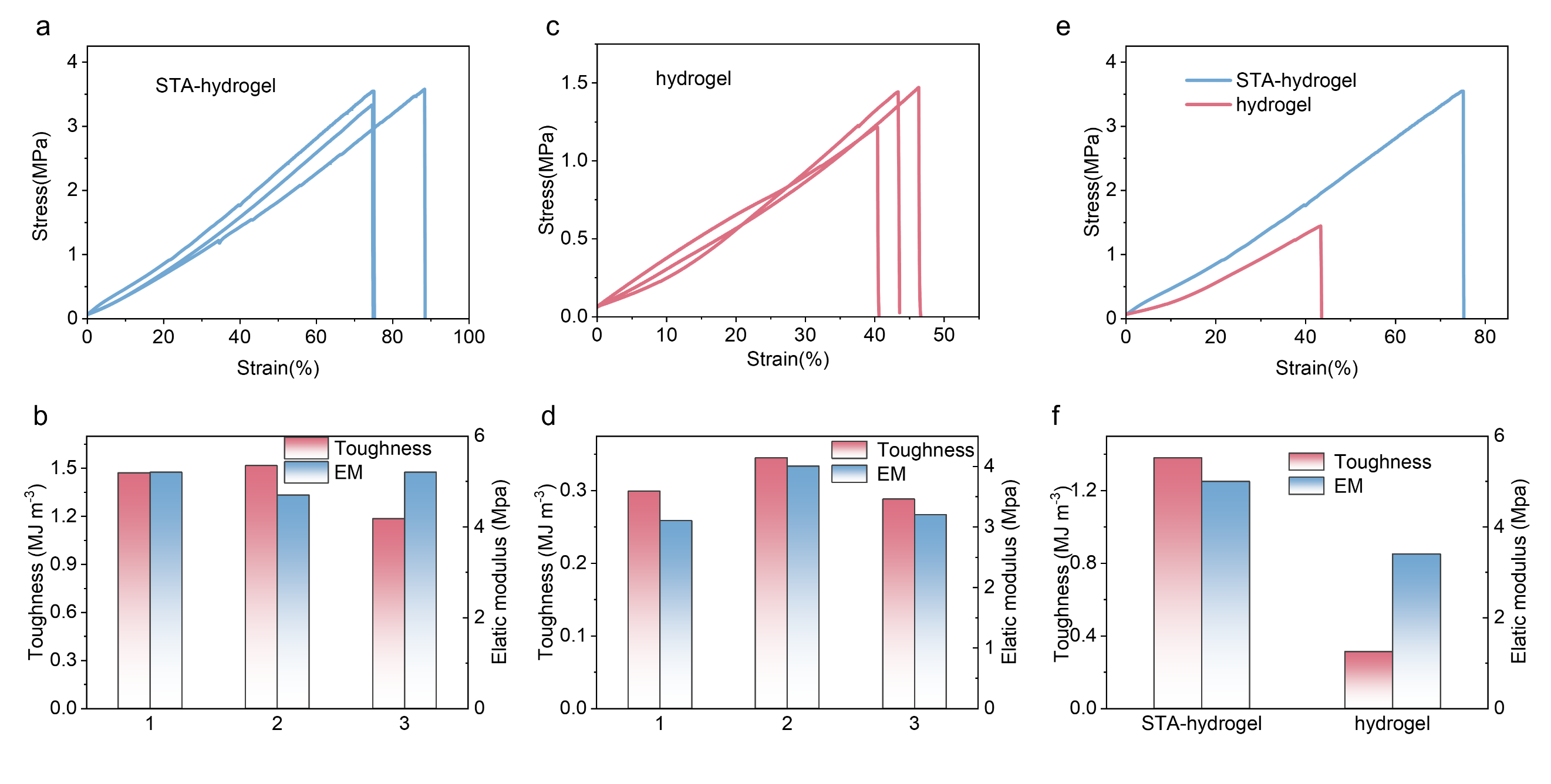


**Figure S9.** Tensile stress-strain curves and the corresponding toughness and elastic modulus. (a) and (b) STA hydrogel, (c) and (d) pristine hydrogel, (e) comparison of two hydrogel, (f) means of two hydrogels.


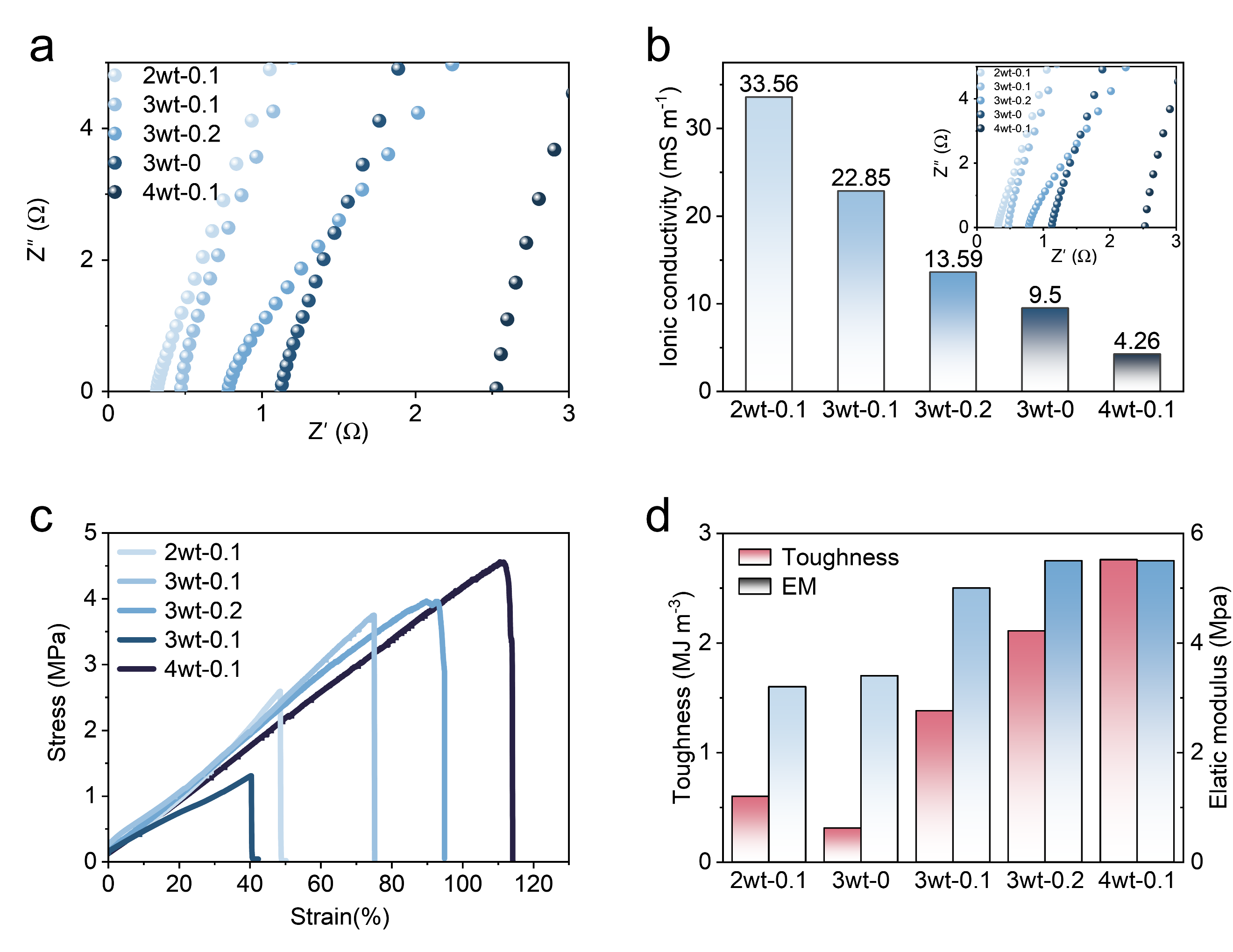


**Figure S10.** Mechanical and electrical properties of hydrogels with different crosslinking densities. (a) The EIS spectra, (b) Nyquist plots and calculated ionic conductivity, (c) Mechanical tensile stress-strain curves, (d) The corresponding toughness and elastic modulus.


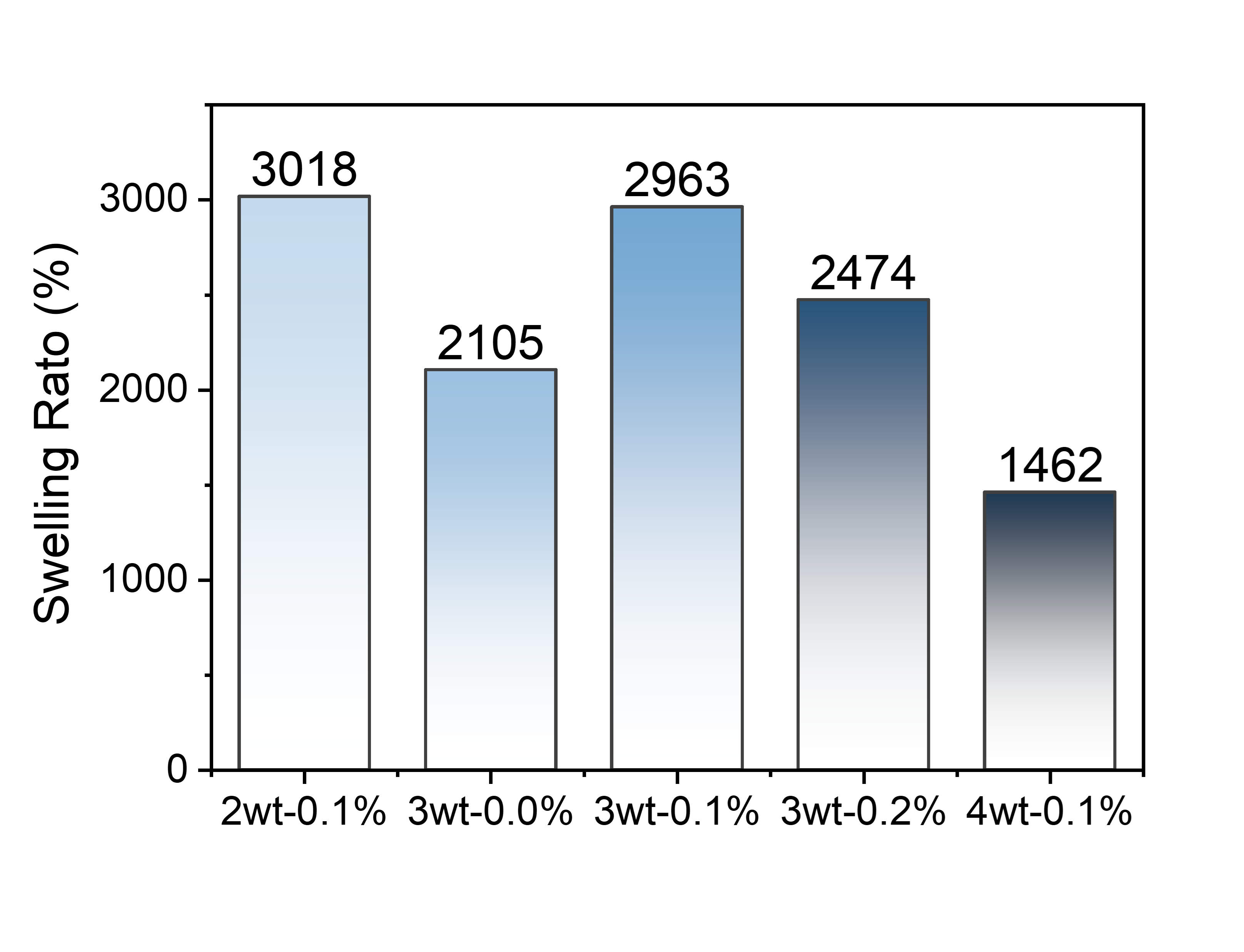


**Figure S11**. Swelling ratios of different hydrogel electrolytes measured as the volumetric expansion from the dry state to the fully swollen hydrogel state.


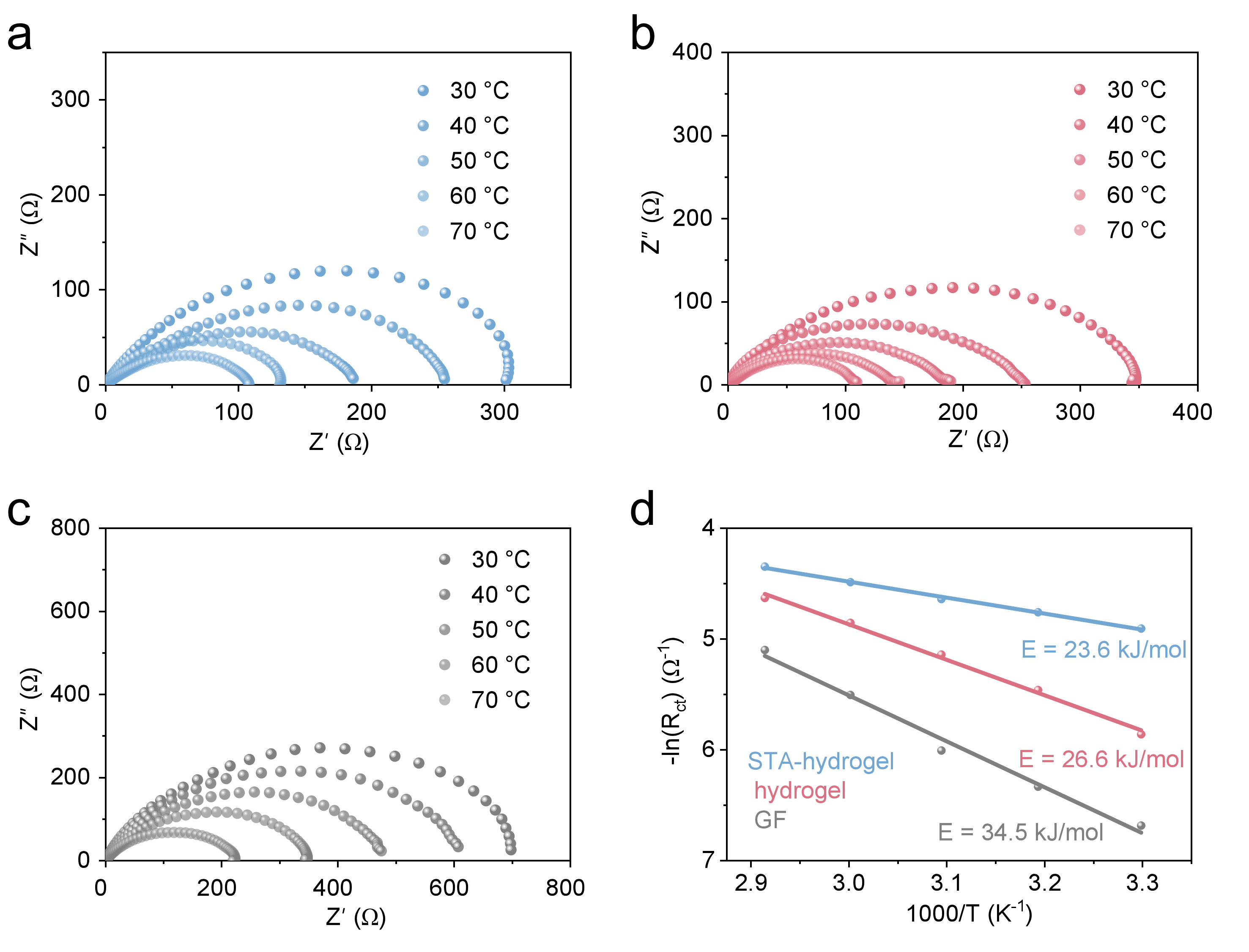


**Figure S12.** Nyquist plots of Zn||Zn symmetrical cell of (a) SAT hydrogel, (b) pristine hydrogel and (c) liquid electrolyte at different temperature. (d) activation energy fitted by Equation (10).


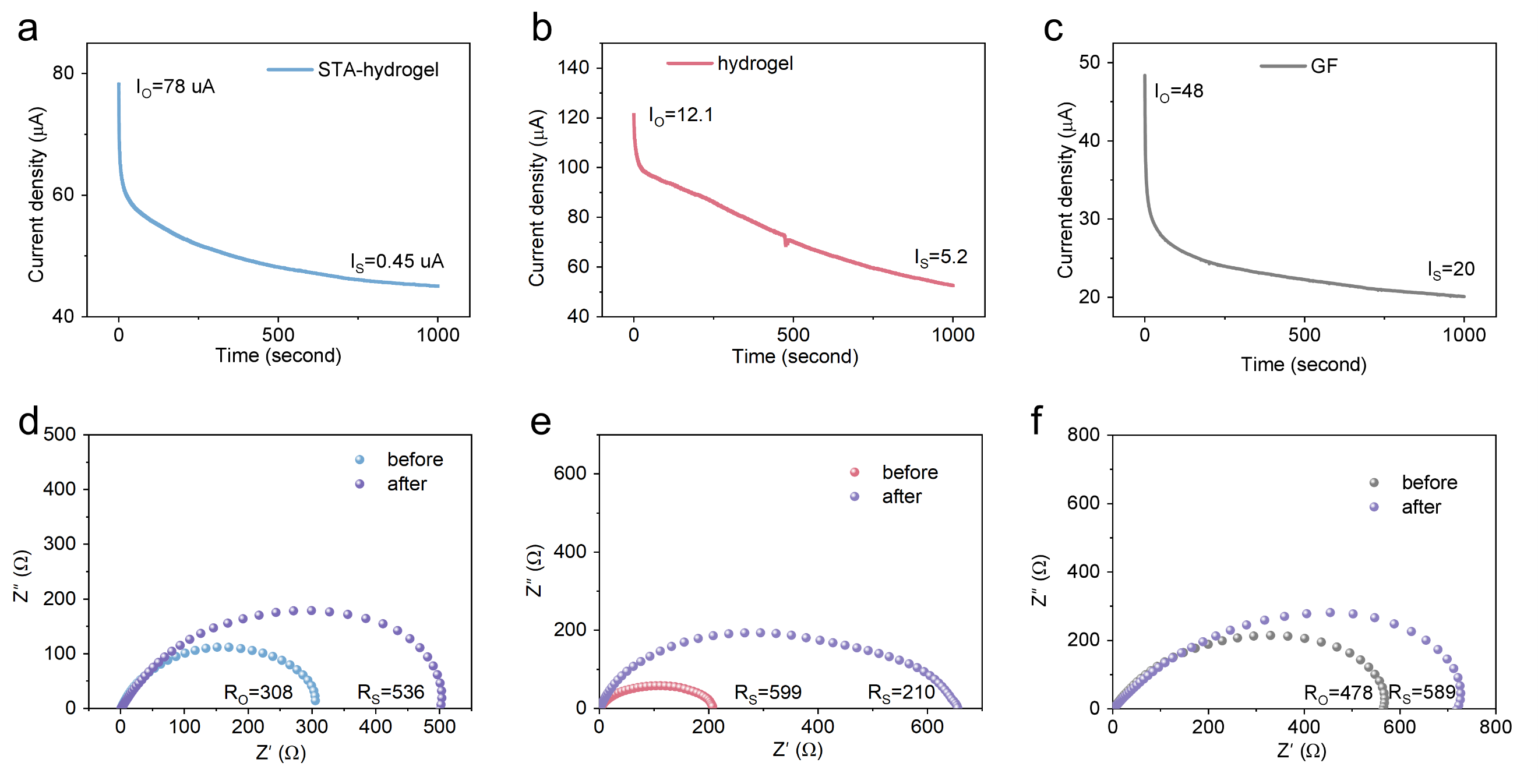


**Figure S13.** Transfer number measurements. Chronoamperometry (CA) curves with the bias voltage set at 40 mV and Nyquist plots of Zn||Zn symmetric cells at the initial and steady states of (a) and (d) STA hydrogel, (b) and (e) pristine hydrogel, (c) and (f) liquid electrolyte.


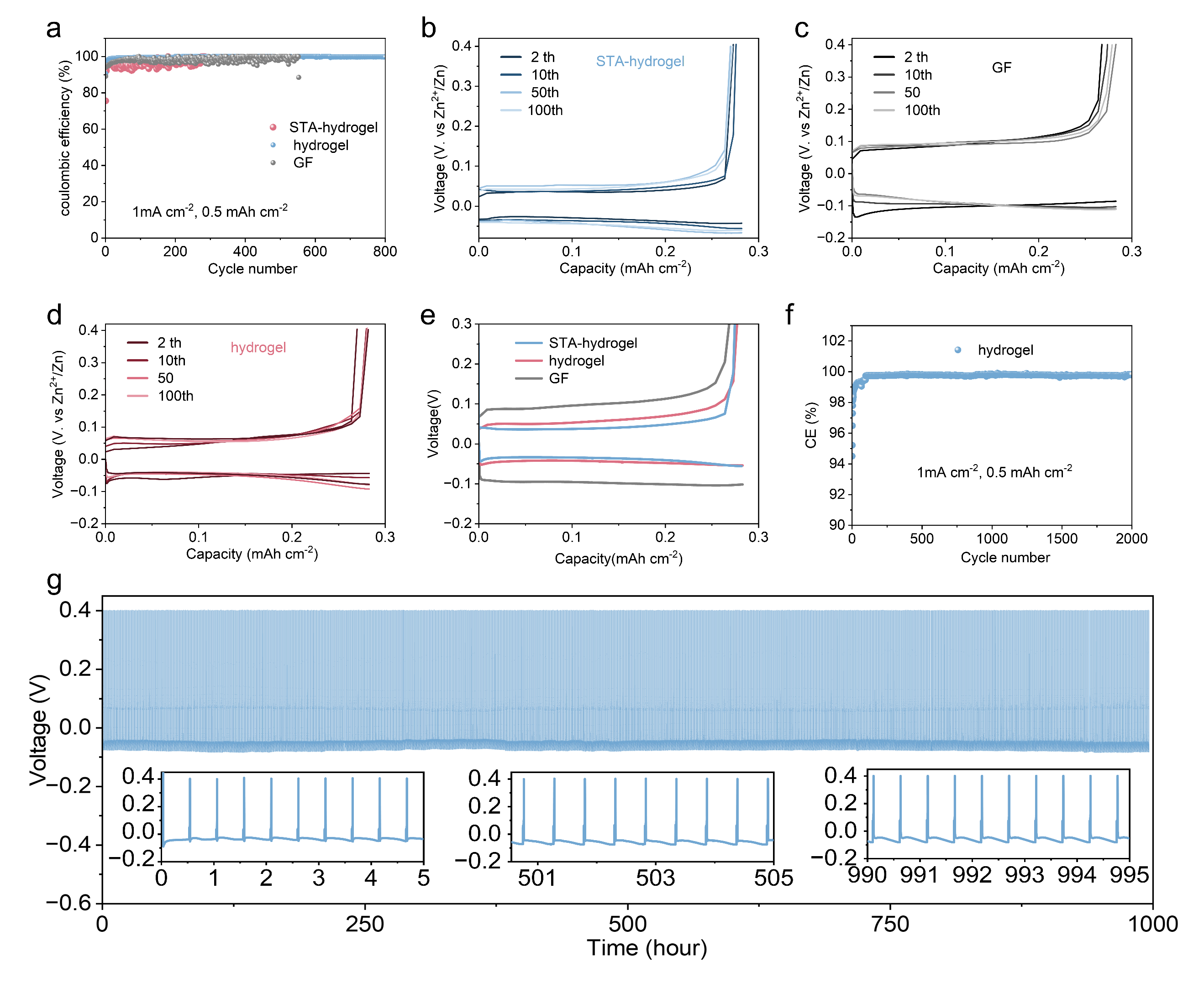


**Figure S14.** (a) The coulombic efficiency of Zn||Cu asymmetric cells with three electrolytes, respectively. Corresponding voltage-capacity curves of Zn||Cu asymmetric cells with (b) STA hydrogel electrolyte, (c) liquid electrolyte, and (d) pristine hydrogel electrolyte, (e) comparison at 50 cycles. (f) Repeated test of CE to demonstrate excellent repeatability, (g) The cycle curve of Zn||Cu asymmetric cells with STA hydrogel electrolyte.


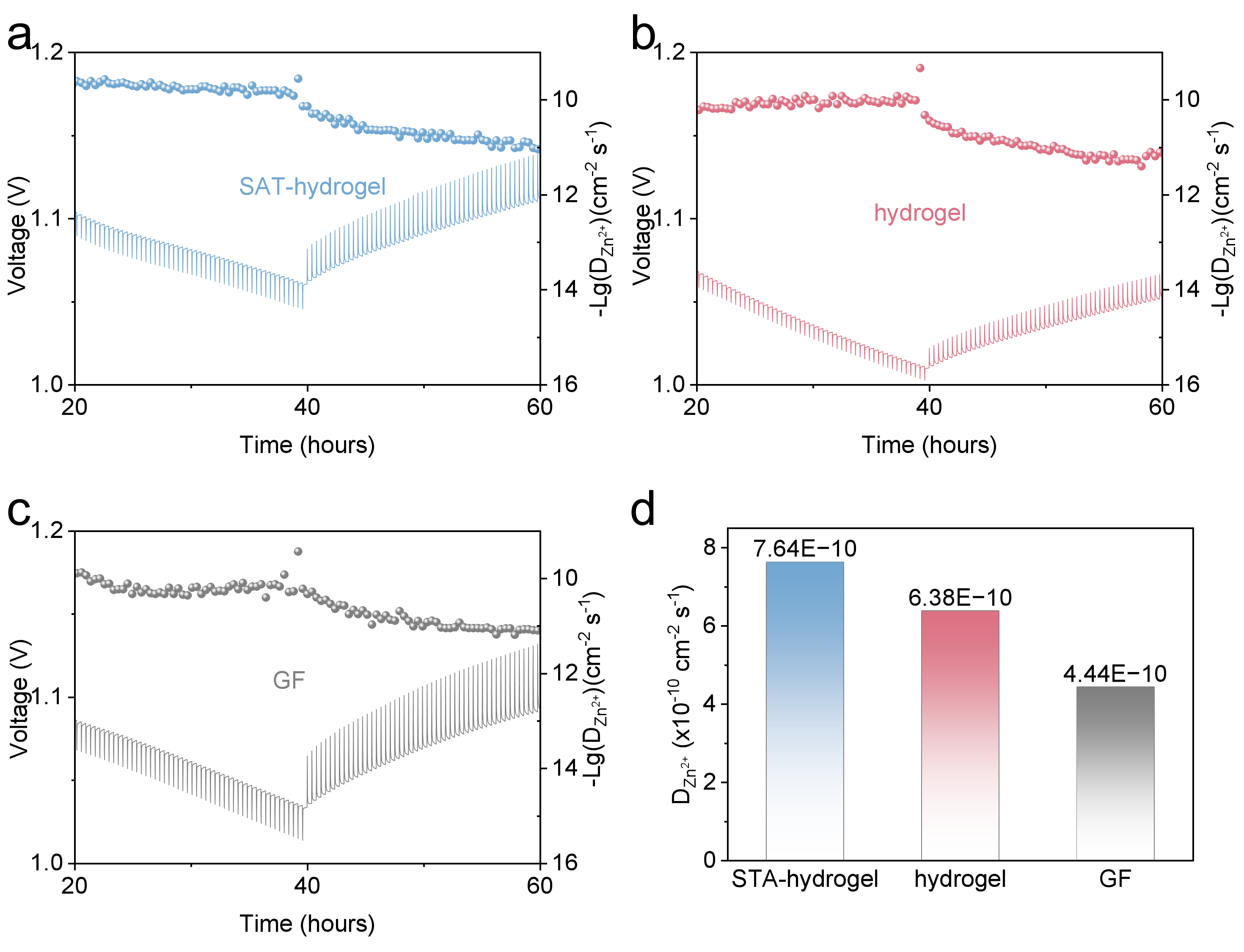


**Figure S15.** GITT curves and corresponding D_Zn_ calculated by Equation (3) of Zn||V_2_O_5_ full cells with (a) STA hydrogel, (b) pristine hydrogel and (c) Liquid electrolyte with glass fiber separator. (d) D_Zn_^2+^ of three electrolytes.


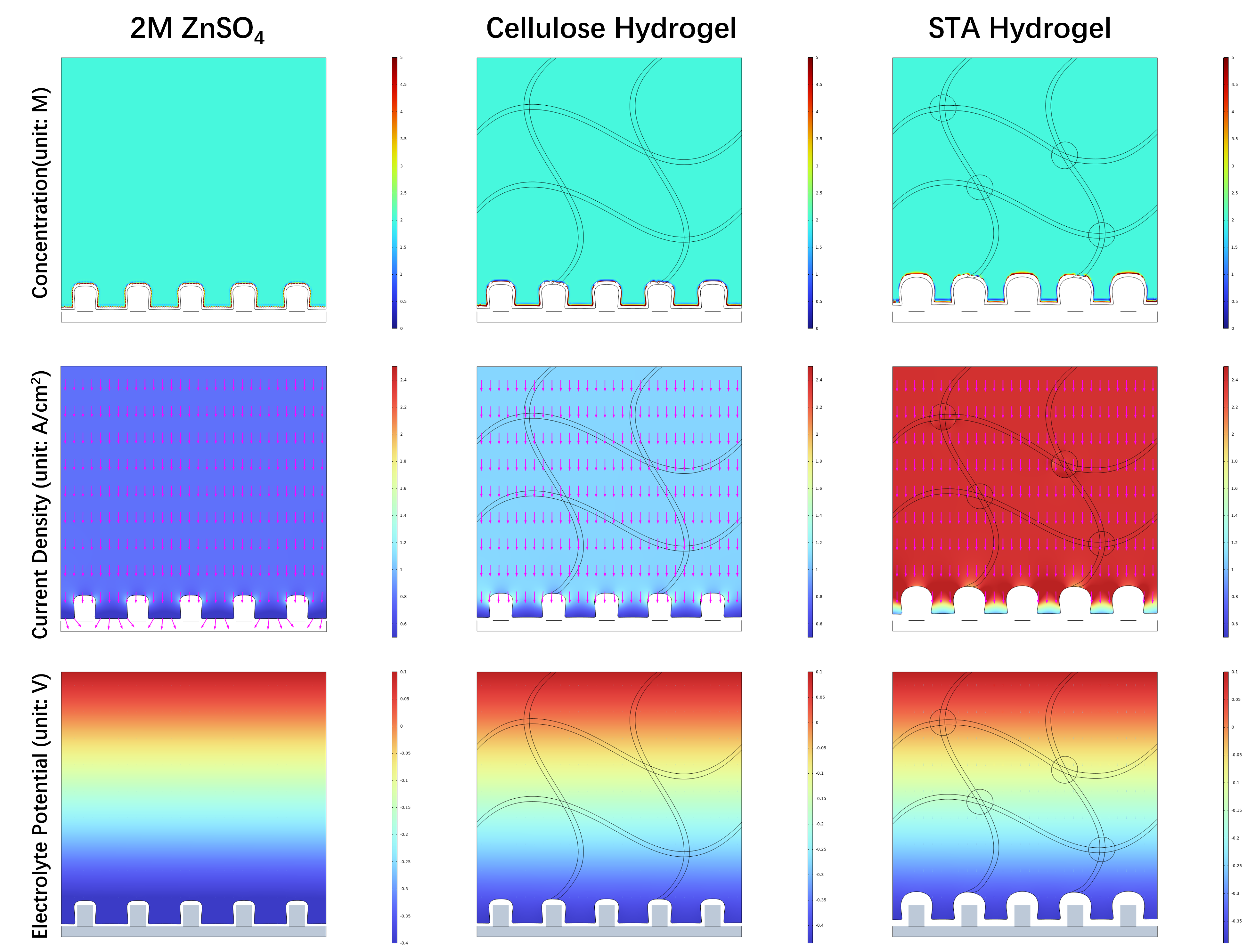


**Figure S16**. Simulated distributions of zinc ion concentration, current density, and electric potential on the Zn electrode surface in different electrolytes.


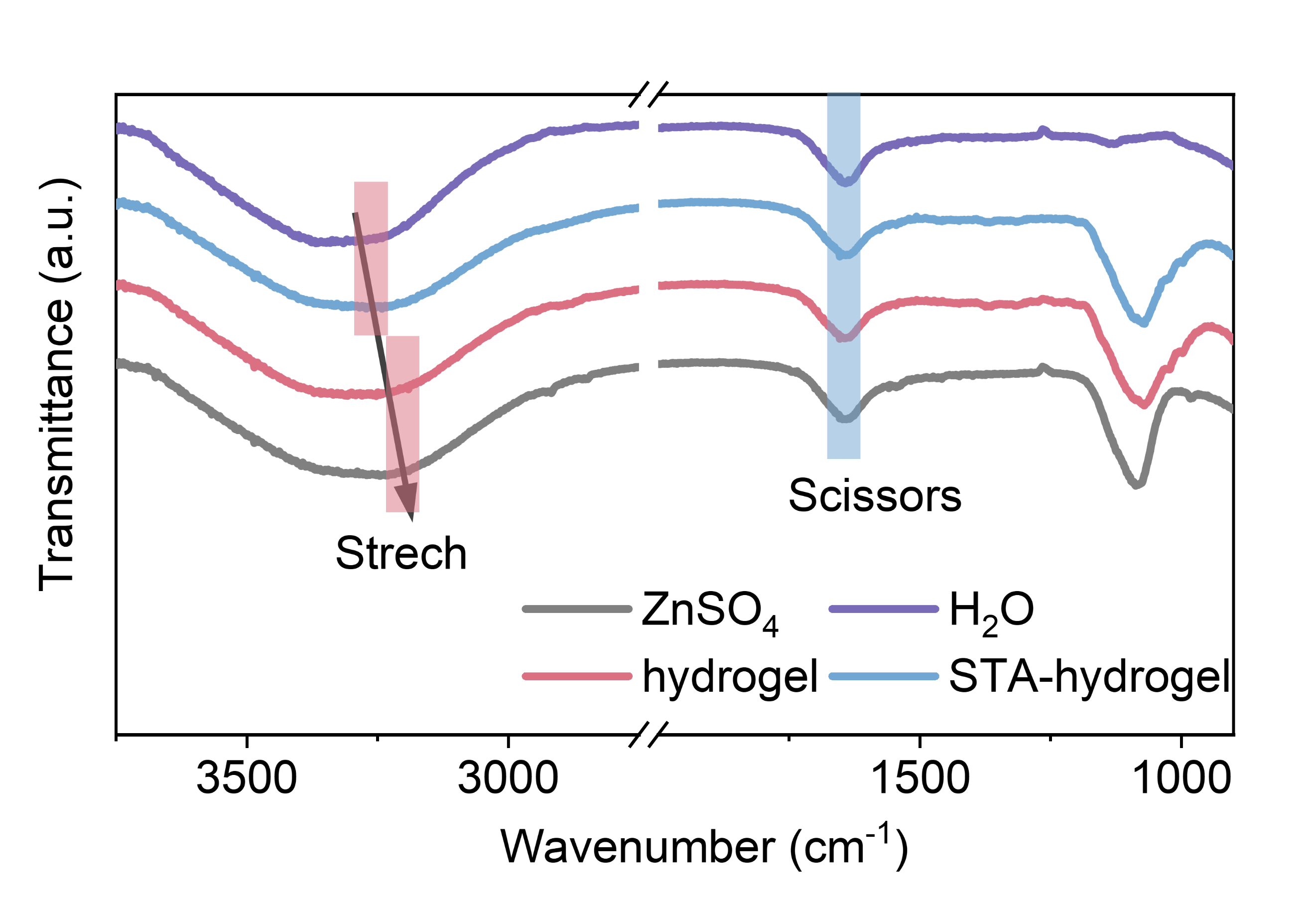


**Figure S17**. FTIR spectra of different electrolytes


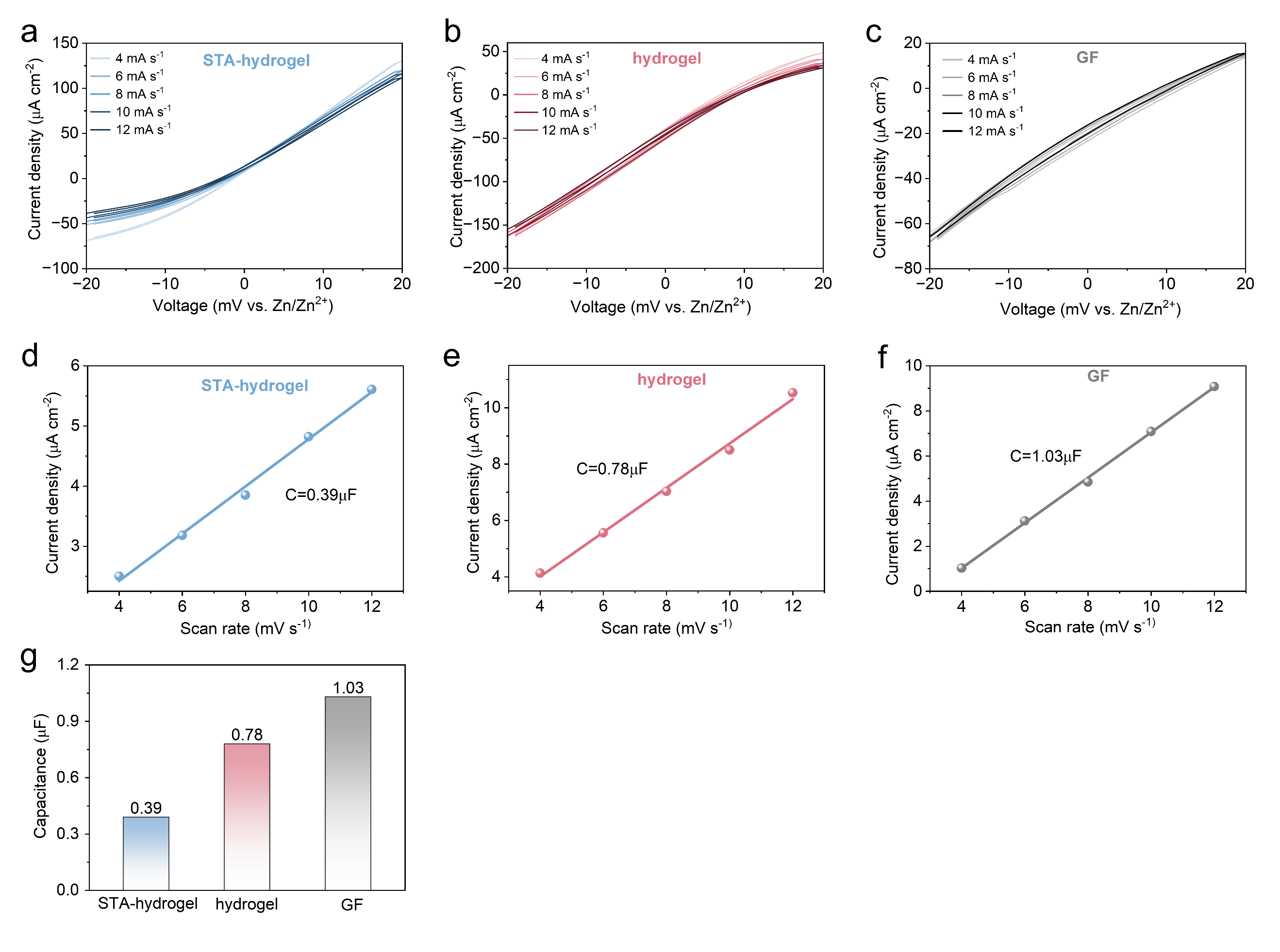


**Figure S18**. EDL measurements for Zn foils in various electrolytes. (a) STA hydrogel, (b) pristine hydrogel and (c) 2M ZnSO_4_ liquid electrolyte with GF separator. Linear fitting of (d) STA hydrogel, (e) pristine hydrogel and (f) 2M ZnSO_4_ liquid electrolyte with GF separator. (g) Capacitance.


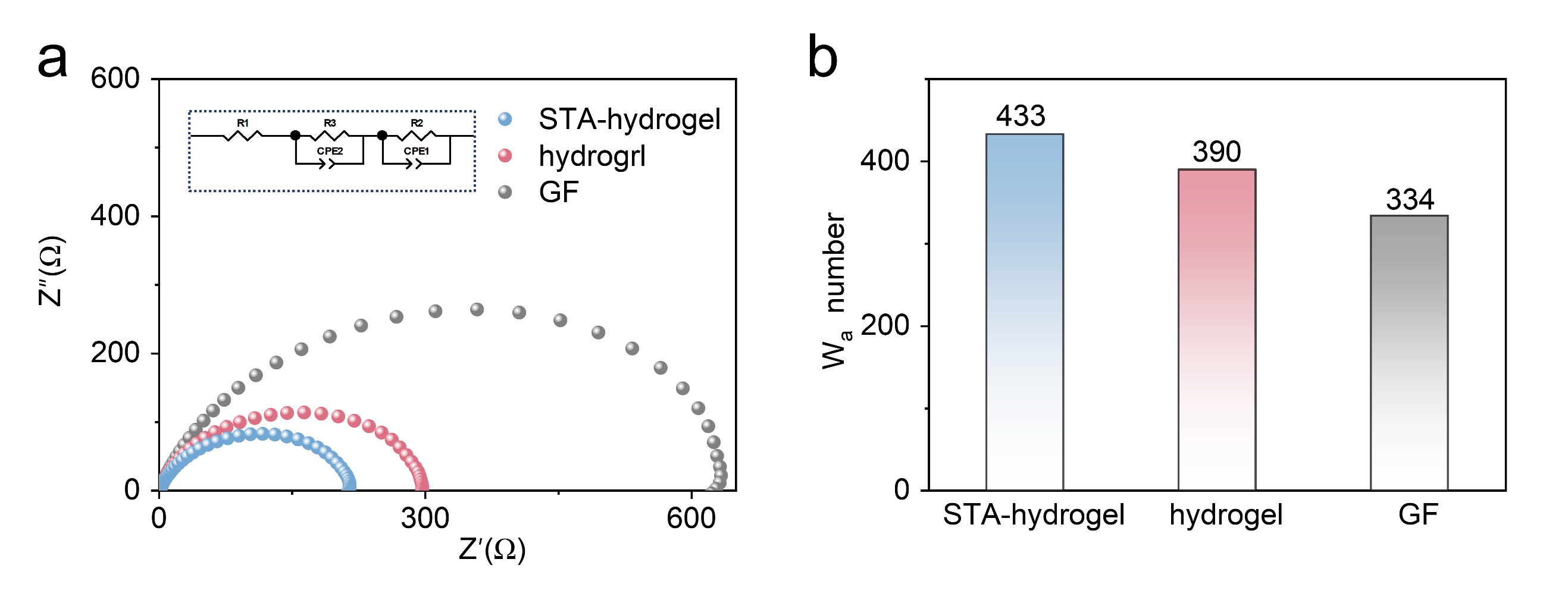


**Figure S19.** (a) Nyquist plots of Zn||Zn symmetrical cell and corresponding equivalent circuit fitting (inset). (b) corresponding *W_a_* number.

**Table 3.** Fitting impedance parameters and *Wa* number

| Electrolyte | R_s_ (Ω) | R_ct_ (Ω) | *Wa* number |
| --- | --- | --- | --- |
| STA hydrogel | 0.493 | 213.6 | 433 |
| Pristine hydrogel | 1.26 | 489.9 | 390 |
| Liquid electrolyte | 1.85 | 619.5 | 334 |


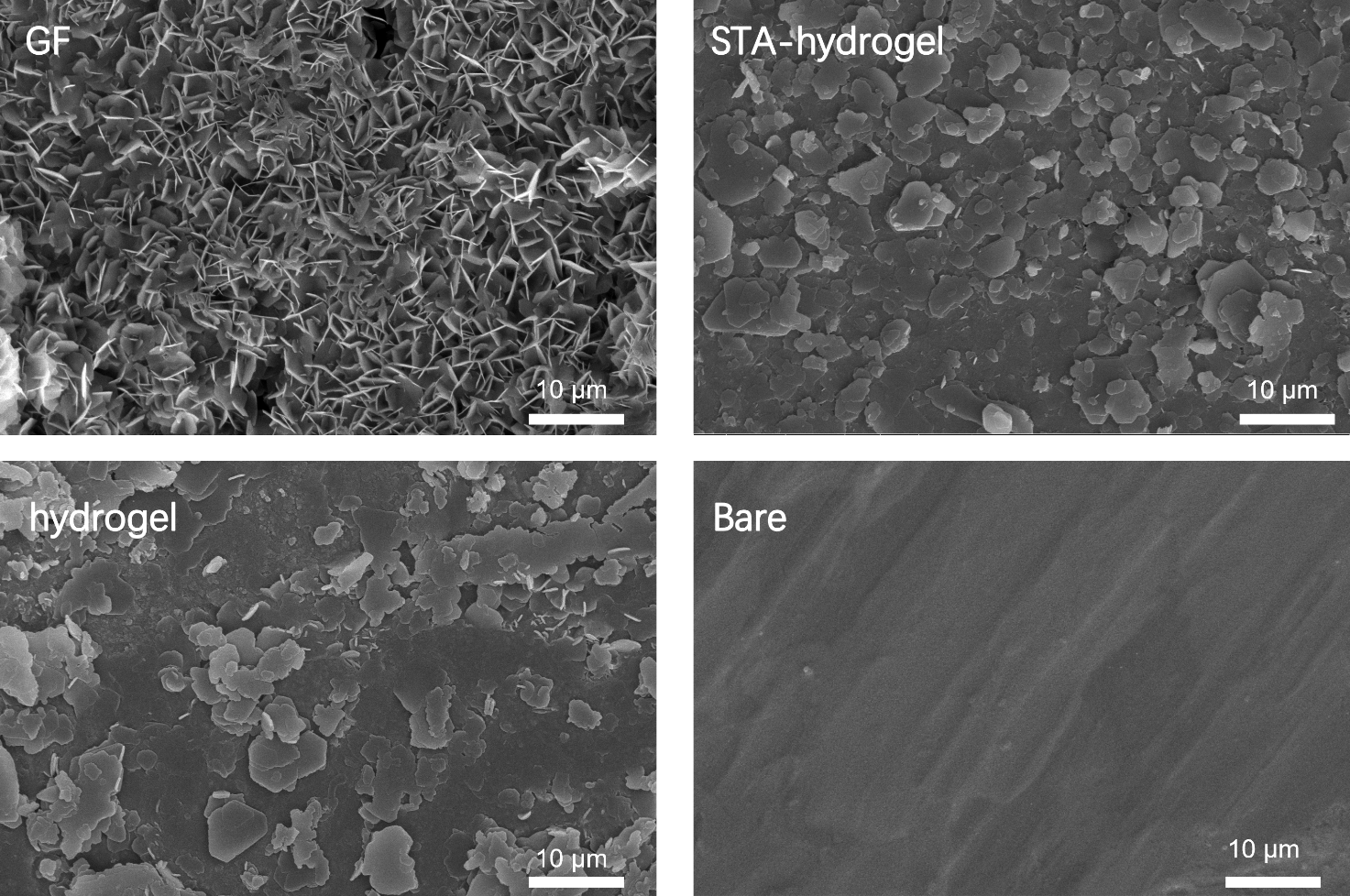


**Figure S20.** Scanning electron microscopy (SEM) images of zinc anode after 50 cycles at a current density of 1 mA cm^-2^ assembled with (a) liquid electrolyte with GF separator, (b) STA hydrogel, and (c) pristine hydrogel. (d) The SEM image of pristine Zn foil.


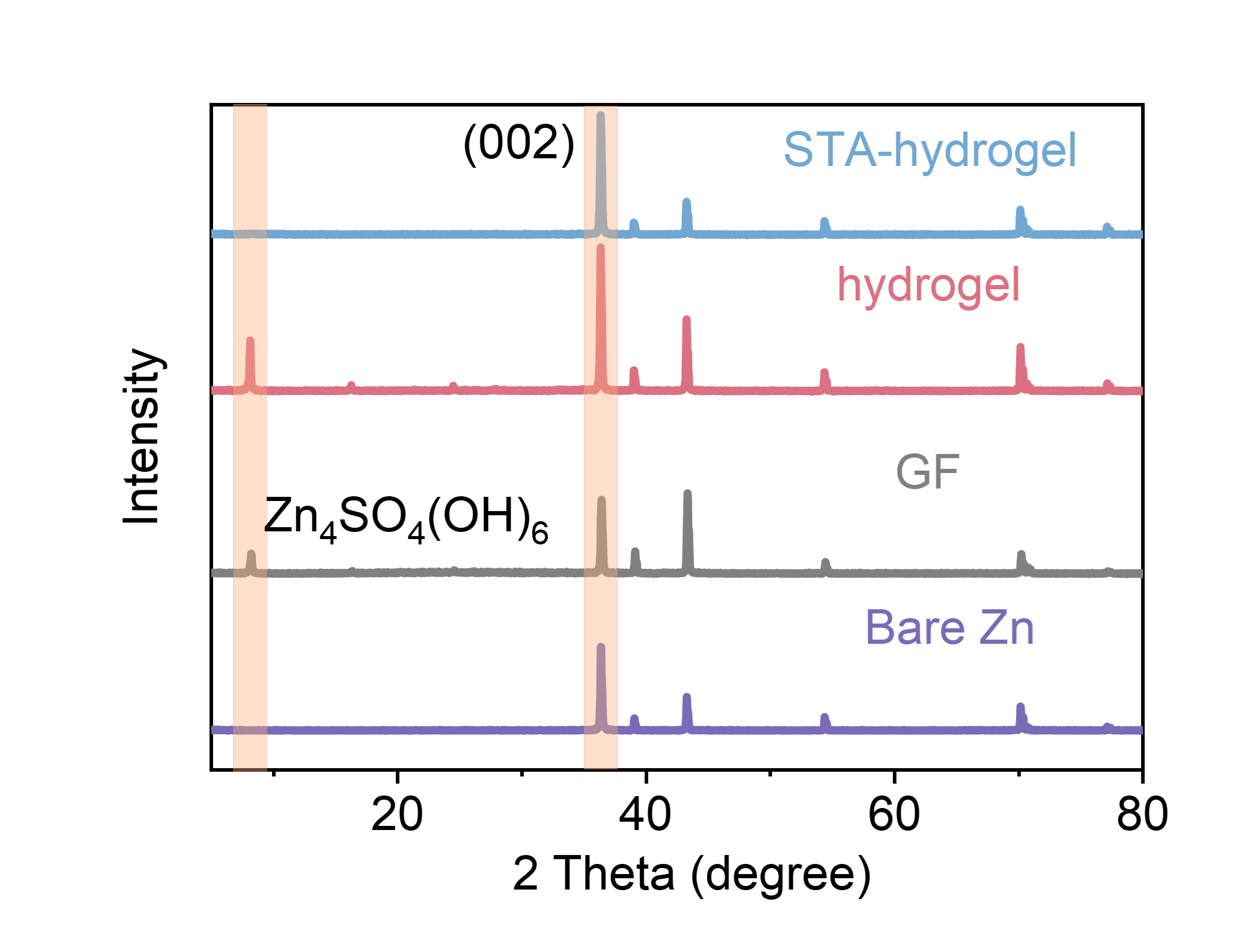


**Figure S21.** XRD patterns of Zn anode after 50 cycles (at 1 mA cm^-2^) in Zn||Zn symmetric cells.


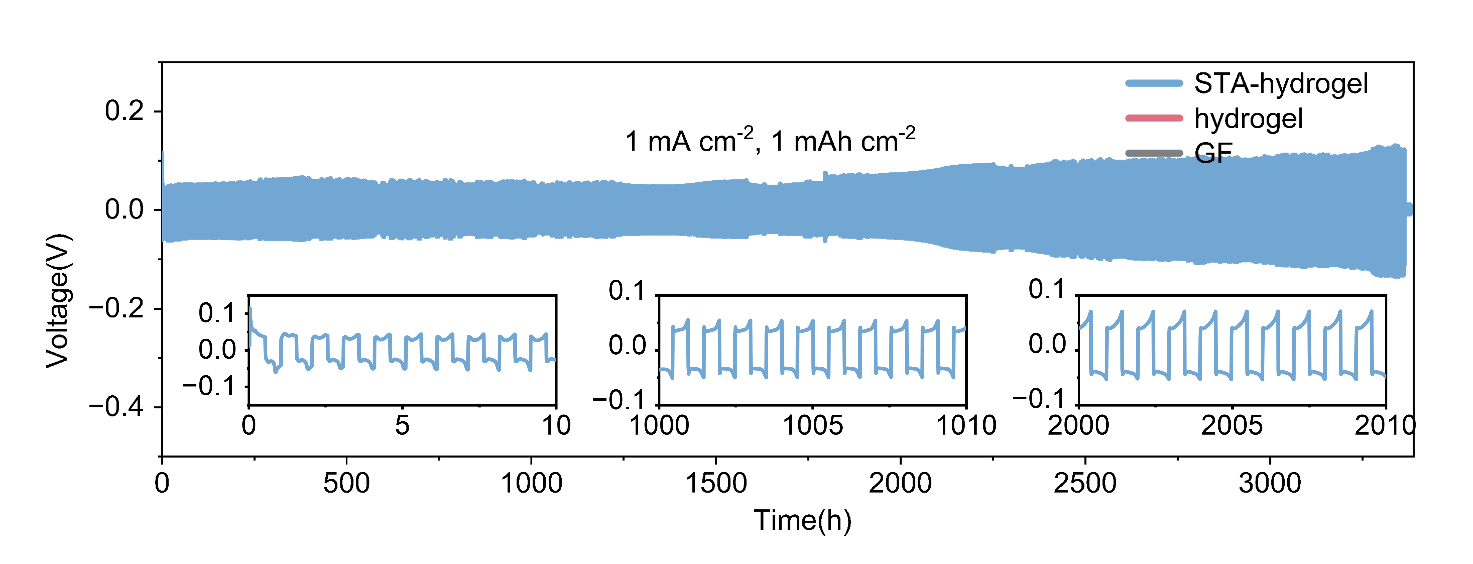

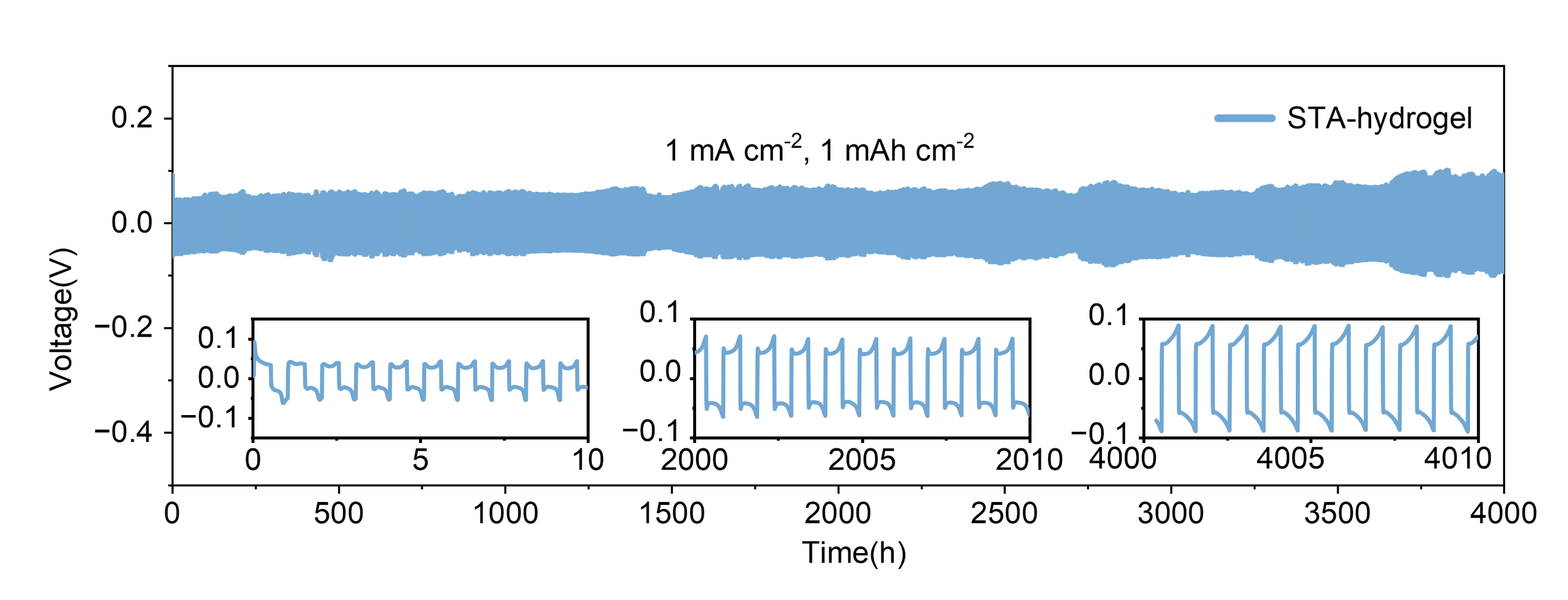


**Figure S22.** Repeated tests of Galvanostatic cycling performance of Zn||Zn symmetric cells at 1 mA cm^-2^ and 1 mAh cm^-2^, to demonstrate excellent repeatability.


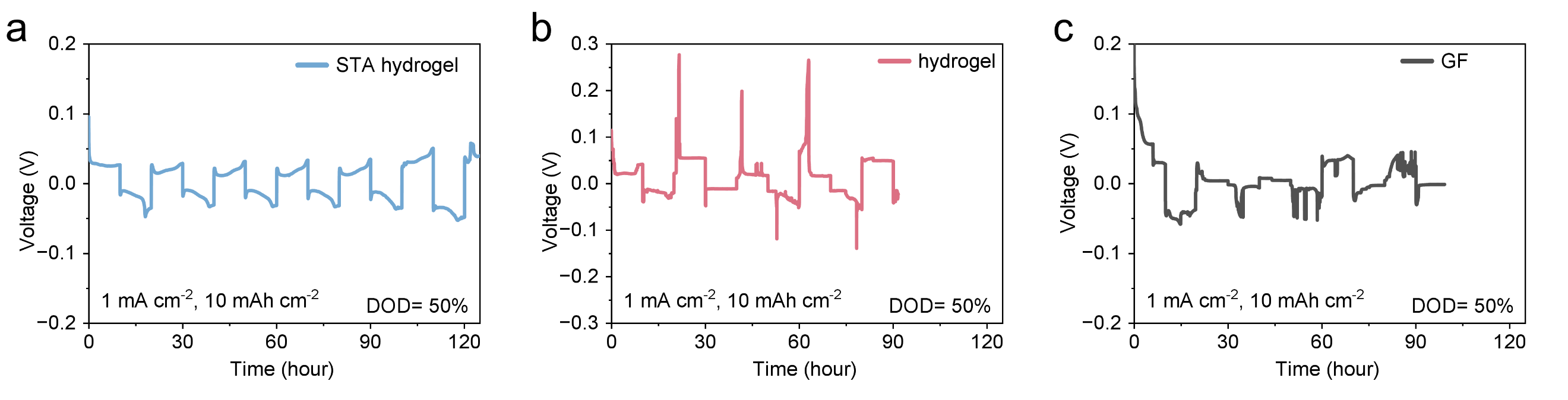


**Figure S23.** Deep discharge curves with depth of discharge 50% (1 mA cm^-2^, 10 mAh cm^-2^) of Zn||Zn symmetric cells, (a) STA hydrogel, (b) pristine hydrogel, and (c) Liquid electrolyte.


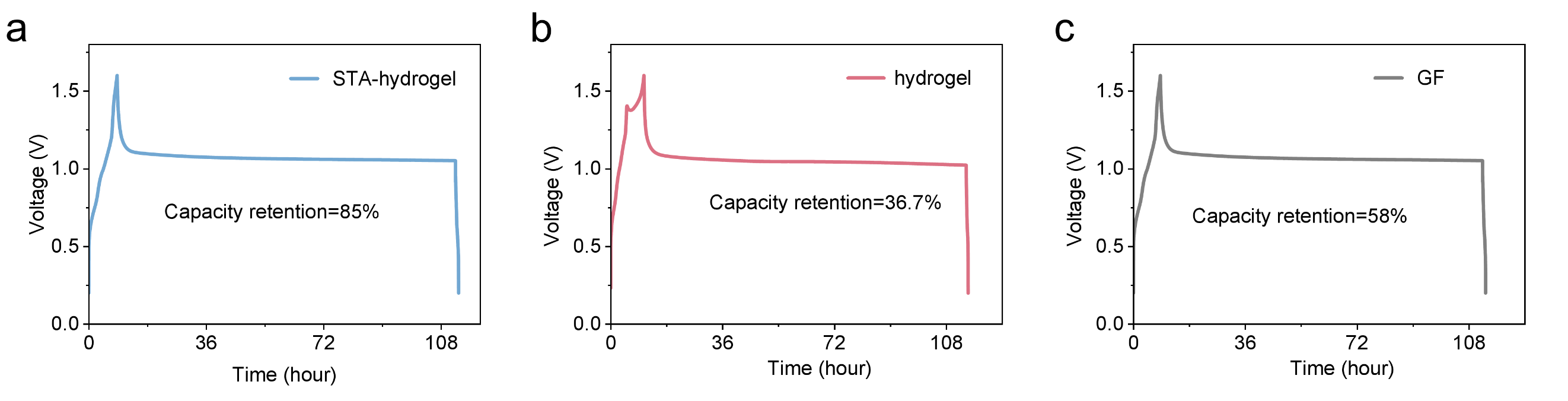


**Figure S24.** Self-discharge curves of Zn||V_2_O_5_ full cells after rest for 72 hours. (a) STA hydrogel, (b) pristine hydrogel, and (c) liquid electrolyte.

**Table S4**. The performance comparison between STA hydrogel electrolyte and other reported cellulose hydrogel electrolyte for aqueous zinc-ion battery.

| Cellulose hydrogel Electrolytes | Solvent | Strength/Elongation at break  (MPa / %) | Ionic conductivity ( mS cm^–1^) | Zn\|\|Zn Cycle life (h)  /current density | Ref. |
| --- | --- | --- | --- | --- | --- |
| **STA hydrogel** | **[TMGH][MAA]** | **3.5/80** | **22.5** | **4200 (1 mA cm^-2^)** | **This work** |
| CT3G30 | NaOH/H_2_O→ZnSO_4_/MnSO_4_/H_2_O | 2.11/846.5 | 32.3 | 800 (2 mA cm^-2^) | 1 |
| DCZ-gel | LiOH/Urea/H_2_O | 2.08/154 | 38.6 | 2000 (0.5 mA cm^-2^) | 2 |
| CE | NaOH/Urea/H_2_O | <0.1/43 | 18.46 | 3000 (1mA cm^-2^) | 3 |
| PCZ-gel | NaOH/Urea/H_2_O | 0.2/990 | 27.5 | 3000 (0.5 mA cm^-2^) | 4 |
| COOH-f-Cell | TMG/DMSO/CO_2_ | 0.22/900 | 10.6 | 2100 (1 mA cm^-2^) | 5 |
| Sor-Cel | NaOH/H_2_O→ZnCl_2_/CaCl_2_/H_2_O | 0.62/303 | 35.4 | 800 (2 mA cm^-2^) | 6 |
| DCG | DBNACl/ 3-Amino-1-propanol | 0.8/17 | 29.5 | 4000 (4 mA cm^-2^) | 7 |
| ZnBr_2_ cellulose hydrogel | ZnBr/H_2_O | 0.019/58 | 29.9 | 11 (3 mA cm^-2^) | 8 |
| Zinc-ion hybrid cellulose supercapacitor | ZnCl_2_/CaCl_2_/H_2_O | 0.37/100 | 74.9 |  | 9 |
| Con-CMC | Water | 1.33/185 | 34.5 | 200 (5 mA cm^-2^) | 10 |
| CMC-GO | Water | 0.00138/70 | 20.5 | 800 (1 mA cm^-2^) | 11 |
| BC-KOH-KI | Bacterial synthesis | 2.1/57 | 54 | 17 (5 mA cm^-2^) | 12 |
| CL gel | alkali–urea solution | 2.0/8 | 330 | 1700 (2 mA cm^-2^) | 13 |
| CPZ-gel | alkali/urea solution | 1.8/80 | 16.4 | 5000 (0.5 mA cm^-2^) | 14 |

**Reference**

1. Chen, M. F.; Chen, J. Z.; Zhou, W. J.; Han, X.; Yao, Y. G.; Wong, C. P. Realizing an All-Round Hydrogel Electrolyte toward Environmentally Adaptive Dendrite-Free Aqueous Zn-MnO_2_ Batteries. *Advanced Materials* **2021**, *33*, 2007559.
2. Zhang, H. D.; Gan, X. T.; Yan, Y. Y.; Zhou, J. P. A Sustainable Dual Cross-Linked Cellulose Hydrogel Electrolyte for High-Performance Zinc-Metal Batteries. *Nano-Micro Letters* **2024**, *16*, 106.
3. Tong, M. D.; Kuang, S. J.; Wang, Q. Y.; Li, X.; Yu, H. X.; Zeng, S. S.; Yu, X. Y. Dual cross-linked cellulose-based hydrogel for dendrites-inhibited flexible zinc-ion energy storage devices with ultra-long cycles and high energy density. *Carbohydrate Polymers* **2024**, *343*, 122444.
4. Zhang, H. D.; Gan, X. T.; Song, Z. P.; Zhou, J. P. Amphoteric Cellulose-Based Double-Network Hydrogel Electrolyte Toward Ultra-Stable Zn Anode. *Angewandte Chemie-International Edition* **2023**, *62*, e202217833.
5. Chen, K.; Huang, J.; Yuan, J. L.; Qin, S. D.; Huang, P. F.; Wan, C.; You, Y.; Guo, Y. L.; Xu, Q. Q.; Xie, H. B. Molecularly engineered cellulose hydrogel electrolyte for highly stable zinc ion hybrid capacitors. *Energy Storage Materials* **2023**, *63*, 102963.
6. Quan, Y. H.; Zhou, W. J.; Wu, T.; Chen, M. F.; Han, X.; Tian, Q. H.; Xu, J. L.; Chen, J. Z. Sorbitol-modified cellulose hydrogel electrolyte derived from wheat straws towards high-performance environmentally adaptive flexible zinc-ion batteries. *Chemical Engineering Journal* **2022**, *446*, 137056.
7. Zhai, J. C.; Zhao, W.; Wang, L.; Shuai, J. B.; Chen, R. W.; Ge, W. J.; Zong, Y.; He, G. J.; Wang, X. H. Ultrathin cellulosic gel electrolytes with a gradient hydropenic interface for stable, high-energy and flexible zinc batteries. *Energy & Environmental Science* **2025**, *18*, 4241.
8. Shi, X.; Yang, L.; Yao, J. Cellulose-based water-in-salt ZnBr_2_ hydrogels with multiple functions for energy storage devices. *Materials Chemistry and Physics* **2024**, *327*, 129923.
9. Yang, L.; Song, L.; Feng, Y.; Cao, M.; Zhang, P.; Zhang, X.; Yao, J. Zinc Ion Trapping in Cellulose Hydrogel as Solid Electrolyte for Safe and Flexible Supercapacitor. *Journal of Materials Chemistry A* **2020**, *8*, 12314-12318.
10. Quan, Y.; Ma, H.; Chen, M.; Zhou, W.; Tian, Q.; Han, X.; Chen, J. Salting-Out Effect Realizing High-Strength and Dendrite-Inhibiting Cellulose Hydrogel Electrolyte for Durable Aqueous Zinc-Ion Batteries. *ACS Applied Materials & Interfaces* **2023**, *15*, 44974-44983.
11. Li, X.; Li, Y.; Jiang, Y.; Wang, D.; Ran, F. Designing carboxymethyl cellulose based hydrogel electrolyte membranes enhanced by inorganic nanoparticle toward stable zinc anode. *Green Energy & Environment* **2025**, *10*, 537-550.
12. Zhang, Y.; Chen, Y.; Li, X.; Alfred, M.; Li, D.; Huang, F.; Wei, Q. Bacterial cellulose hydrogel: A promising electrolyte for flexible zinc-air batteries. *Journal of Power Sources* **2021**, *482*, 228963.
13. Shi, X.; Dong, C.; Ma, L.; Yang, M.; Chen, X.; Zhang, Y.; Liu, L.; Long, Z. A sustainable and recyclable cellulose gel electrolyte enables stable zinc metal anode for green aqueous batteries. *Chemical Engineering Journal* **2025,** *504*, 158659.
14. Zhang, H.; Gan, X.; Gao, Y.; Wu, H.; Song, Z.; Zhou, J. Carboxylic Acid‐Functionalized Cellulose Hydrogel Electrolyte for Dual‐Interface Stabilization in Aqueous Zinc‐Organic Batteries. *Advanced Materials* **2025,** *37*, 2411997.
